# The SLE Transcriptome Exhibits Evidence of Chronic Endotoxin Exposure and Has Widespread Dysregulation of Non-Coding and Coding RNAs

**DOI:** 10.1371/journal.pone.0093846

**Published:** 2014-05-05

**Authors:** Lihua Shi, Zhe Zhang, Angela M. Yu, Wei Wang, Zhi Wei, Ehtisham Akhter, Kelly Maurer, Patrícia Costa Reis, Li Song, Michelle Petri, Kathleen E. Sullivan

**Affiliations:** 1 The Division of Allergy Immunology, The Children's Hospital of Philadelphia, Philadelphia, Pennsylvania, United States of America; 2 The Center for Biomedical Informatics, The Children's Hospital of Philadelphia, Philadelphia, Pennsylvania, United States of America; 3 Department of Computer Science, New Jersey Institute of Technology, Newark, New Jersey, United States of America; 4 Division of Rheumatology, Johns Hopkins University School of Medicine, Baltimore, Maryland, United States of America; National Cancer Institute, National Institutes of Health, United States of America

## Abstract

**Background:**

Gene expression studies of peripheral blood mononuclear cells from patients with systemic lupus erythematosus (SLE) have demonstrated a type I interferon signature and increased expression of inflammatory cytokine genes. Studies of patients with Aicardi Goutières syndrome, commonly cited as a single gene model for SLE, have suggested that accumulation of non-coding RNAs may drive some of the pathologic gene expression, however, no RNA sequencing studies of SLE patients have been performed. This study was designed to define altered expression of coding and non-coding RNAs and to detect globally altered RNA processing in SLE.

**Methods:**

Purified monocytes from eight healthy age/gender matched controls and nine SLE patients (with low-moderate disease activity and lack of biologic drug use or immune suppressive treatment) were studied using RNA-seq. Quantitative RT-PCR was used to validate findings. Serum levels of endotoxin were measured by ELISA.

**Results:**

We found that SLE patients had diminished expression of most endogenous retroviruses and small nucleolar RNAs, but exhibited increased expression of pri-miRNAs. Splicing patterns and polyadenylation were significantly altered. In addition, SLE monocytes expressed novel transcripts, an effect that was replicated by LPS treatment of control monocytes. We further identified increased circulating endotoxin in SLE patients.

**Conclusions:**

Monocytes from SLE patients exhibit globally dysregulated gene expression. The transcriptome is not simply altered by the transcriptional activation of a set of genes, but is qualitatively different in SLE. The identification of novel loci, inducible by LPS, suggests that chronic microbial translocation could contribute to the immunologic dysregulation in SLE, a new potential disease mechanism.

## Introduction

Systemic lupus erythematosus (SLE) is the quintessential systemic autoimmune disease. The etiopathogenesis is still not fully understood and there are over 20,000 published studies evaluating various aspects of cellular dysfunction in this disease. Over the past 10 years, insights have come from genome-wide association (GWA) studies as well as gene expression studies. In both cases, a type I interferon pathway was implicated [1], [2].

One of the hallmarks of lupus is the presence of autoantibodies directed against nucleic acid targets and other nuclear antigens. The process of apoptosis exposes the immune system to nucleic acids and nuclear antigens, particularly when the apoptotic cells are not cleared appropriately and degrade into smaller components [3]. The apoptotic debris are believed to drive much of the type I interferon signature and the type I interferon itself can drive additional apoptosis. Nevertheless, much remains unknown about the pathogenesis of SLE, particularly at the level of nucleic acid accumulation and dysregulated gene expression. Dysregulated gene expression, with the accumulation of aberrant transcripts, could theoretically contribute to apoptosis or increased type I interferon expression and has been shown to mimic lupus [4], [5], [6], [7], [8], [9].

We utilized next generation sequencing of transcripts (RNA-seq) to characterize the SLE transcriptome in monocytes. Monocytes are a critical cell in SLE. They are implicated in renal damage, which is the major cause of morbidity in SLE, and in atherosclerosis, which is the major cause of mortality in SLE [10], [11], [12], [13]. Monocytes are, therefore, central to the disease process, but are also of interest because they respond to environmental stimuli, alter their function accordingly, and reflect that information back to other immunologically competent cells. They offer the additional advantage of representing a relatively homogeneous population [14]. This is the first RNA-seq study of SLE and we found not only a transcriptome that exhibits quantitative alterations as defined by the level of gene expression, but also qualitative differences with widely altered splicing preferences and non-coding RNA transcription. Some novel transcripts expressed at higher abundance in SLE monocytes were inducible by LPS, known to activate type I interferons [15], [16], [17]. LPS and microbial products have been demonstrated to accelerate renal disease and induce lupus-like processes in mice [18], [19], [20], [21]. This finding provides an additional perspective from which to understand SLE.

## Methods

### Patients and cell purification

Investigators at Johns Hopkins University (JHU) School of Medicine obtained written informed consent and HIPAA Authorization of study subjects for all SLE samples. The Institutional Review Board at Johns Hopkins reviewed and approved the study of SLE patients. The use of the anomyzed Red Cross samples was approved by the Red Cross Institutional Review Board. Control samples were obtained from The Center For Aids Research, which supplies blood samples on a fee for service basis. They have obtained consent for the use of the samples and their protocols were approved by the University of Pennsylvania Institutional Review Board.

Primary human monocytes were purified using elutriation and adherence from eight healthy controls and nine SLE patients with no other autoimmunity, as previously described [22], [23], [24], [25]. The purity of monocytes was more than 90% by flow cytometry for CD14 staining. All controls and patients were female and an average of approximately 40 years of age. All SLE patients' disease activity was mild-moderate and no one was on high-level immune suppression (Table S1). All subjects were enrolled in an IRB-approved study and provided informed consent, except the red cross serum samples which were provided as anomyzed discarded samples.

### RNA isolation and library preparation

Total RNA was isolated from 2–3 million primary monocytes using the Qiagen RNeasy kit and DNA was removed by on-column DNase digestion (Qiagen, Valencia, CA). This method recovers predominantly RNA species >200 bases. 1 µg of total RNA was used to prepare the library with the SOLiD™ whole transcriptome analysis kit (Applied Biosystems, Foster City, CA), providing strand-specific data. The procedure followed the instructions of the manufacturer. For miRNA validation, we used miRNeasy from Qiagen. Table S2 provides RNA quality and cell count information. The OD 260/280 ratio ranged from 1.8–1.98. RIN scores were not consistently obtained prior to fragmentation but were >7 for those where the RIN was defined. Post fragmentation, the RIN score averaged 2.3. The RNA quality and counts were not different between patients and controls.

### RNA abundance validation

qRT-PCR was used to define quantitative differences in RNA abundance. The Clontech Advantage RT for PCR kit (Clontech, Mountain View, CA) was used to generate cDNA. Gene expression was detected by real-time PCR using the TaqMan 9600. Transcript levels were normalized to the 18S or β-actin signal, as has been previously used in SLE studies [26], [27], [28]. Mature miRNAs were detected with Taqman miRNA assays. Relative quantitation was performed using spiked *Caenorhabditis elegans* miRNA-238 as an exogenous control (Qiagen Syn-cel-miR-238-3p miScript miRNA Mimic). Commercially available primers were purchased from Applied Biosystems for: cel-miR-238 (248 primer for isoform cel-miR-238-3p - MIMAT0000293); hsa-miR-212 (515 primer for isoform hsa-miR-212-3p - MIMAT0000269 and 461768_mat for isoform hsa-miR-212-5p - MIMAT0022695); and *CCR2* (Hs00174150 primer for *CCR2* isoform NM_001123041.2 and Hs00704702_s1* for *CCR2* isoform NM_001123396.1) and from Qiagen for *RND3* (QT00002744),*TSLP* (QT01670809), *RGPD1* (QT01678425), *CD177* (QT02452849), *TUBB1* (QT00049574), and *ITG-B* (QT01003121). Novel loci were detected with custom primers using SYBR green. Primer sequences are listed in the Methods S1. The Mann Whitney U test was used to analyze the differences between SLE and controls.

### Endotoxin analyses

MonoMac 6 cells and primary monocytes from healthy donors were stimulated with 100 U/ml α2-interferon (PBL Biomedical Laboratories, Piscataway, NJ), 10 ng/ml γ–interferon (R&D Systems, Inc., Minneapolis, MN), 10 ng/ml tumor necrosis factor (TNF-α) (Sigma, Saint Louis, MO) for 16 hours, or 1 µg/ml of LPS for two hours. SB203580 was used as a p38- MAPK inhibitor by pretreating the cells for 30 minutes at a concentration of 10 µM. SP600125 (Calbiochem, Darmstadt, Germany) was used as a JNK inhibitor at a concentration of 10 µM and U0126 (Cell signaling, Danvers, MA) was used as an ERK inhibitor at a concentration of 10 µM. Cells were harvested after stimulation and RNA was prepared as above. To measure circulating endotoxin, serum samples from 99 SLE patients and 112 Red Cross blood donors were analyzed using the Limulus assay (Thermo Scientific, Rockford, IL). The Wilcoxon method was used to compare the levels across groups.

### Bioinformatics

We used the Tophat-Cufflinks pipeline and further refinements [29] to assemble the monocyte transcriptome and detect novel loci and isoforms, followed by mapping short reads to a collection of reference RNA sequences, including isoforms of coding genes, small RNAs, long non-coding RNAs (lncRNAs), and repetitive elements. The number of reads mapped to each transcript was used for evaluating differential expression between control and SLE samples. Data has been submitted to GEO as GSE53419.

The following steps were used in data analysis:

- Use TopHat to align 50 bp sequencing reads to reference genome hg19. TopHat also searched for reads partially mapped to distant locations to identify exon-exon junctions. As a result, TopHat mapped ∼22 million reads per sample on average and stored the aligned reads in BAM files. This step did not use any known gene annotation
- Use Cufflinks to assemble transcriptomes of individual samples while using RefSeq gene annotation as reference. On average, Cufflinks reported 65,008 genes and 83,430 transcripts in individual transcriptomes.
- Use Cuffmerge to combine individual transcriptomes, also using RefSeq as reference. The merging improved the reliability of novel loci and isoforms by taking a consensus of individual transcriptomes. Cuffmerge reported 59,887 transcripts of 34,307 genes in a consensus transcriptome.
- Use Cuffcompare to categorize transcripts by comparing the consensus transcriptome to RefSeq annotation. For example, a class “ = ” transcript had the same exon-exon junctions as a known RefSeq transcripts and a class “u” transcripts had no overlapping to any known genes. As a result, Cuffcompare identified 10,200 novel transcribed loci and 10,313 isoforms including novel exon-exon junctions of known genes.
- Use the gene annotation tracks downloaded from UCSC Genome Browser to evaluate the novelty of the new loci and isoforms reported by Cuffcompare. The tracks were UCSC Genes, Ensembl Genes, GENCODE Genes V12, CCDS, H-Inv 7.0, sno/miRNA and lincRNA Transcripts, including evidence-based gene annotation, computationally generated gene prediction, and collections of non-coding RNAs. The comparison identified 2,280 and 4,000 high novelty loci and isoforms not included in any of the seven annotation tracks.
- Repeat the TopHat-to-Cuffcompare steps without using a reference annotation for assembling and merging transcriptomes. Cuffcompare identified 774 transcripts having the exact same exon-exon junctions as known RefSeq transcripts. These true positives had higher read counts and consistency across samples. Surprisingly, they were also longer and included more exons on average, which presumably made them more difficult to be completely assembled. High read count and high consistency were then used as the criteria to filter the novel loci and isoforms identified by previous steps to obtain 3,725 and 1,327 high-confidence novel loci and isoforms respectively.

#### Align reads to reference transcriptome using Novoalign

NovoalignCS was used to align reads to a reference transcriptome to obtain the read count per transcript. Each entry of the reference transcriptome was the full sequence of a transcript without introns. The reference transcriptome included all RefSeq transcripts and novel loci/isoforms reported by Cuffmerge, as well as three classes of non-coding RNAs: small RNAs from snoRNABase and miRBase), lincRNAs (UCSC Genome Browser lincRNAs track), and repetitive elements (Repbase).

Reads mapped to the same transcript were counted to define the transcription level. Reads mapped to different RNA classes or transcripts of different genes were excluded. The number of unique reads mapped to different transcripts of the same genes were used as gene-level read count. Only reads mapped to the sense strand were counted, whereas reads mapped to the antisense strand of coding genes were used to quantitate antisense transcription.

#### Statistical analysis of transcriptional data

We noticed that the fold change of patients and controls was slightly dependent on the baseline expression level (r = 0.15, p<0.01), and applied Loess adjustment to remove this bias by assuming an equal amount of total RNA between samples. The global average of patient-control difference was very close to zero (0.4% higher in SLE) after the adjustment.

Statistical analysis of differential expression was performed within R environment using Bioconductor packages. Rsamtools package was used to import aligned reads from BAM files to R environment. Gene-level differential expression between SLE and control samples was analyzed with the Bioconductor EdgeR package. Genes without at least three read counts in at least three samples were excluded.

Gene-gene correlation analysis was performed by the following steps: calculate the correlation coefficients between genes in control and SLE groups separately; convert correlation coefficients to z scores using Fisher's transformation; take the average of z scores, and convert the average z score back to a single combined correlation coefficient r. Enrichment studies utilized DAVID which reports adjusted p values based on the Benjamini Hochberg algorithm [30].

#### 3′ Untranslated analysis

For analysis of the 3′ untranslated regions, RNA-Seq reads were aligned to reference genome (UCSC, hg19) using Tophat 1.3.1 by default parameters for Applied Biosystems' Colorspace format. Uniquely mapped reads were kept for downstream analysis. PolyA_DB together with the 3′ ends of transcripts from UCSC, RefSeq, Aceview annotations were collected as a comprehensive APA database [31]. For each annotated cleavage site, the number of reads in the upstream 200 nt window were computed as the estimation for the expression level of the mRNA isoform terminating at that 3′end. The read count for each isoform was added up in nine case samples and eight control samples, respectively. We required at least 10 reads supporting each isoform resulting in ∼6600 APA genes for analysis. The relative ratio of expression at proximal to distal sites, in addition with a p-value, was calculated by Fisher's Exact test using R (http://www.r-project.org/). Relative ratio>2 and p-value<0.01 were used to predict different isoform usage.

#### Analysis of published gene sets

Five data sets were created from four monocyte GEO data series, GSE15219, GSE19627, GSE5504, and GSE21909 (one from each of the first three and two from GSE21909, which used saline and cortisol for intravenous delivery of LPS). Samples and groups were selected so that each group had matched replicates/donors. (Table 1)

**Table 1:** GEO sources.

	# groups	# replicates
GSE15219	4	2
GSE19627	2	3
GSE5504	4	2
GSE21909_Saline	3	3
GSE21909_Cortisol	3	3

## Results

### Transcriptome characterization

Monocyte transcriptomes from Nine female SLE patients and eight female healthy controls were studied using RNA-seq. Total RNA was purified from monocytes and used for library preparation. Our first goal in this study was to reconstruct the human monocyte transcriptome using the entire dataset. The Tophat-Cufflinks pipeline reported four major classes of transcripts (Figure S1A), including 10,313 isoforms of known genes and 10,200 transcribed loci not included in the RefSeq annotation. The processed data are given in an excel spreadsheet in Data S1. The novel isoforms had a more complex structure (Figure S1B). On the other hand, the novel loci were shorter on average than known genes and over 97% of them included only one exon.

To further define the monocyte transcriptome, we counted the total number of reads solely aligned to each of the four major RNA classes: coding RNAs, long non coding (lncRNAs), small RNAs, and repetitive elements. Ribosomal RNAs (rRNA) were excluded from this summary. Close to 80% of the non-rRNA reads were mapped to coding RNAs while the other three classes evenly shared the remaining reads (Figure S1C). Small RNAs had the highest molar concentration after read counts were adjusted by the total length of the RNA classes, followed by repetitive elements, coding RNAs, and lncRNAs (Figure S1D). These data were comparable to the distribution seen in other systems [32].

The classifications of transcripts were refined. We identified 4,000 high-novelty isoforms that were not present in seven additional annotated databases and included at least one unknown exon-exon junction, as well as 2,271 high-novelty loci that had no overlap with either exons or introns of any previously reported transcripts. We then defined 1,327 high-confidence isoforms, having at least 10 uniquely mapped reads not mapped to any other genes or isoforms of the same gene in at least six samples, and 3,725 high-confidence novel loci having at least 10 uniquely mapped reads in at least six samples and more than 10× overall sequencing depth. After this refinement, 448 novel isoforms and 778 novel loci that had both high-novelty and high-confidence were identified. There was no association between high-novelty and high-confidence (Figure S2). We manually examined a subset of the 778 novel loci for protein coding potential and the possibility that they are orthologs of coding genes in other species using BLAST. Most did not exhibit similarity to any annotated genes and the majority had limited protein coding potential (<100 contiguous amino acids). We selected 27 novel loci and validated transcription from all 27 using RT-PCR (Figure S3, Table S3).

### Class-specific transcripts in SLE

**Figure 1:**
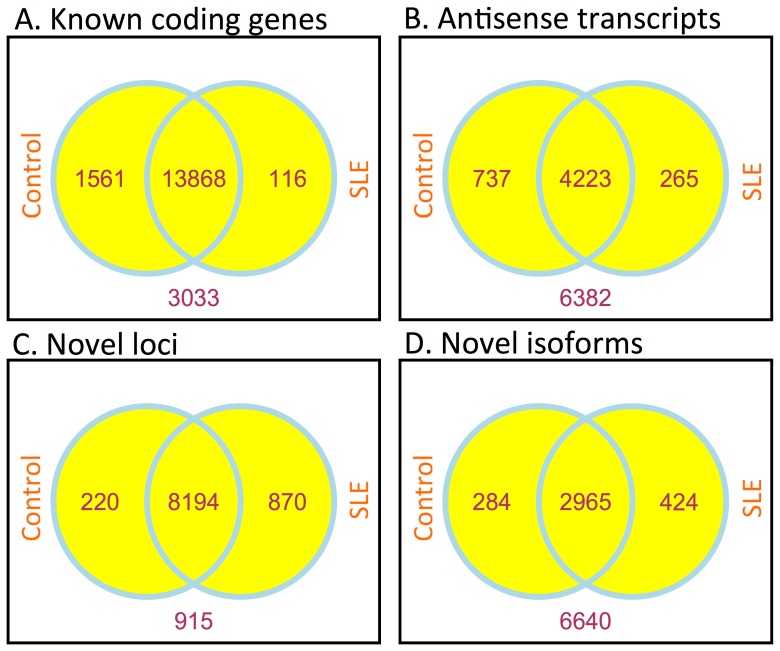
Comparison of SLE and control transcriptomes. The Venn diagrams demonstrate the overlap of actively transcribed A) coding genes, B) antisense transcripts, C) novel loci, and D) novel isoforms in the two sample groups. Eight control and nine SLE libraries were used.

The major goal of this study was to identify a unique signature of the monocyte transcriptome in SLE as a strategy to improve our understanding of the pathogenic mechanisms. We found that there were a large number of known protein coding genes expressed in normal monocytes, but silenced in SLE (Figure 1A). These genes were highly enriched with ones related to embryo development (p = 6.0E-60), suggesting that SLE monocytes are more differentiated. Antisense transcripts were also more likely to be silenced in SLE (Figure 1B). On the other hand, the numbers of novel loci and novel isoforms transcribed only in patients were 3.95 and 1.49 times higher, respectively, than the numbers of control-specific ones (Figure 1C and 1D).

We evaluated the differential expression between the two groups of samples in six classes of genes/transcripts: known RefSeq protein coding genes, novel loci identified by Cufflinks, lncRNAs, small RNAs, repetitive elements, and the antisense transcripts of coding genes. These genes/transcripts were filtered by a statistical analysis, so all the remaining 33,760 (72% of 47,171 total reads) were mapped by at least three sequencing reads in at least three samples. We applied the negative binomial test implemented by the edgeR package of Bioconductor [33] and identified 1,754 differentially expressed genes/transcripts with p values less than 0.01 and a false discovery rate (FDR) of 0.2. Classes of differentially expressed genes (DEGs) are shown in Table S4.

**Figure 2:**
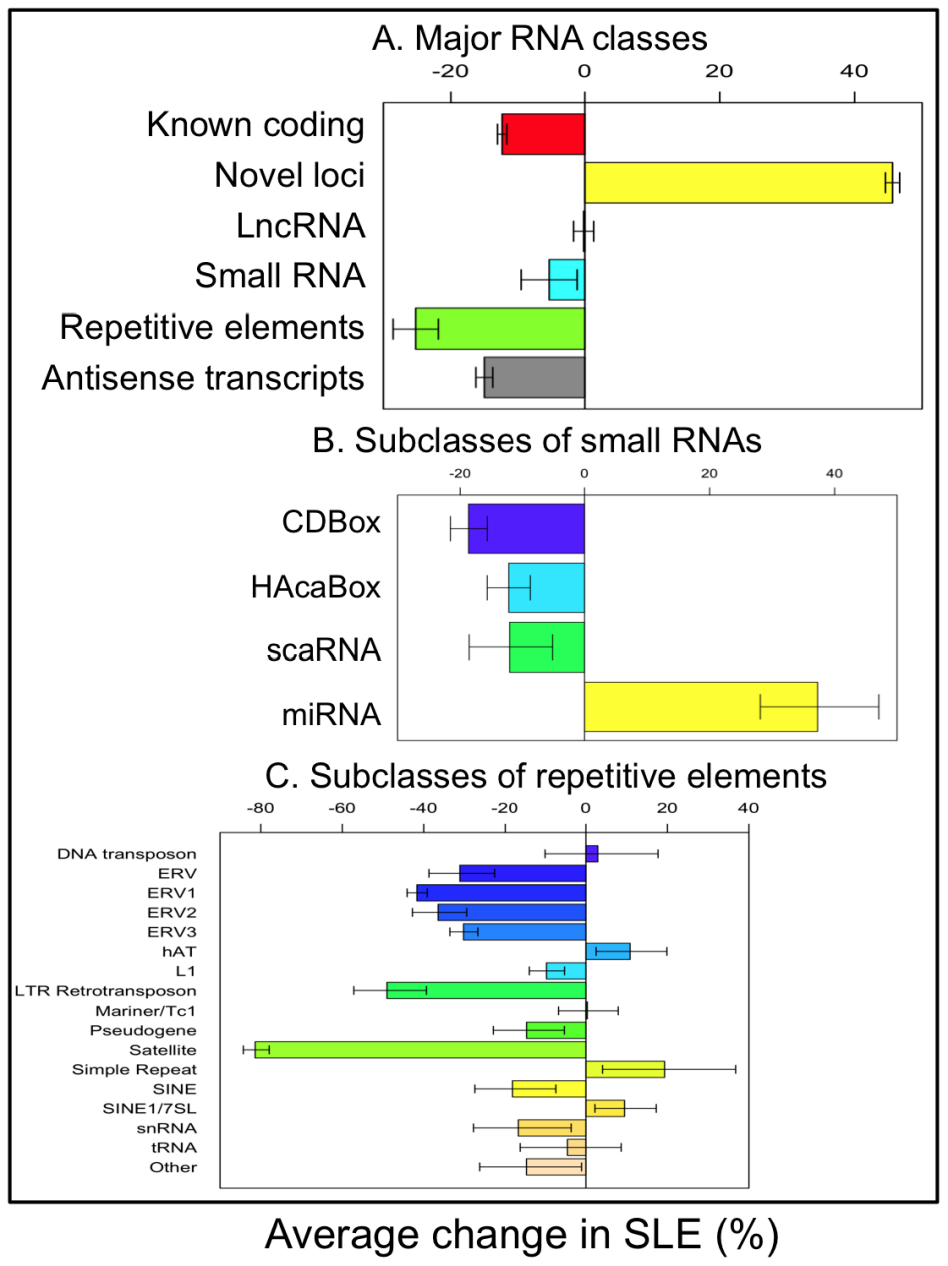
Differential expression of RNA classes. A) The average transcriptional changes of five RNA classes in SLE. B) The average transcriptional change of four sub-classes of small RNAs in SLE. C) The average transcriptional change of 17 subclasses of repetitive elements in SLE. The eight control and nine SLE libraries were used for this analysis. Error bars indicate standard deviation.

The patient-control difference of total transcription varied dramatically between RNA classes (Figure 2A). Total transcription of both sense and antisense transcripts of known coding genes was reduced by approximately 15% in SLE (p = 5.6E-61 and 7.4E-105 respectively). Total transcription of novel loci was strikingly increased by over 45% in SLE (p<1.0E-300), as SLE patients produced many previously undiscovered transcripts that had low or no transcription in healthy monocytes. Although expression of small RNAs was downregulated by about 5%, expression of 91 pri-miRNAs was dramatically increased by 38% (p = 0.01), whereas the three classes of small nucleolar RNAs were all downregulated in SLE patients (Figure 2B). The overall up-regulation of pri-miRNAs and down-regulation of coding genes jointly suggested a modified miRNA regulatory system in SLE. Subclasses of repetitive elements also had different directions of change in SLE (Figure 2C). All endogenous retroviral (ERV) subclasses were consistently downregulated (p = 4.7E-4 to 3.8E-29). Other retrotransposons such as LTR retrotransposons, SINE, and LINE (L1) elements were variably decreased in SLE patients, while expression of SINE1/7SL elements was increased.

### Protein-coding genes

Functional analysis of the protein coding genes identified a large number of pre-defined gene sets whose members were enriched within differentially expressed genes (DEGs), partially listed in Table S5. Most noticeably, DEGs upregulated in SLE were enriched with genes related to immune response and cytokine activity, while the downregulated DEGs were enriched with genes related to cell adhesion and motion. According to Genetic Association Database (GAD), eleven upregulated DEGs were associated with SLE in previous studies (Table S6). In addition, the combined gene set of up- and downregulated DEGs was enriched with potential targets of transcription factors highly relevant to SLE, including AP1 (p = 3.4E-09), E47 (p = 1.1E-8), RFX1 (5.8E-7), IRF1 (p = 1.4E-3), and IRF2 (p = 1.3E-3). We selected six DEGs, five upregulated and one downregulated in SLE, to be validated in new samples using qRT-PCR. Five of the genes were significantly different between controls and patients (p = 0.049 to 0.0001) and all six were changed in the same direction in both patient cohorts (Figure S4).

### Polyadenylation

Polyadenylation along with mRNA cleavage and termination are regulated in a gene-specific manner and these steps appear to be particularly important for the regulation of genes involved in inflammation [34], [35], [36]. Tissue-specific and developmentally-regulated polyadenylation have been described with differentiation favoring longer 3′ UTRs [37], [38], [39]We examined the structure of the coding genes by defining the 3′ untranslated region for each gene. Sixty-seven genes had longer 3′UTRs in patients compared to controls and 54 genes had shorter 3′UTRs in patients (defined by a ratio of >2.0 and a p<0.01). The longer 3′UTR gene set was characterized by pathways centered on NFκB, Akt, UBC and HNF4A. The shorter 3′UTR gene set was characterized by pathways centered on UBC, NFκB, and ERK (Figure S5). As shorter 3′UTRs can escape regulation by miRNAs and the inflammatory pathways implicated by the pathway analysis are concordant with what is known about SLE, these gene sets are notable and may given insight into the SLE process [38], [40].

### Antisense transcription

We analyzed antisense transcription because of the potential for sense-antisense duplex RNA to drive type I interferon expression and because antisense transcripts can regulate transcription in *cis* [41]. The gene-level read counts of sense and antisense transcripts were positively, but weakly, correlated (r = 0.19, p<0.01). Overall, examining patients and controls together, the total number of reads mapped to the antisense transcripts was about 1/30 of the total number of reads mapped to the sense transcripts. However, a small set of genes demonstrated much higher antisense transcription than sense transcription (Figure S6), such as *USP5*, a ubiquitin peptidase, and *CMTM5*, a chemokine-like factor gene.

In SLE, some coding genes had significantly changed antisense transcription in the opposite direction of their sense counterparts, most noticeably *IVNS1A BASES* (influenza virus NS1A binding protein), *RACGAP1* (Rac GTPase activating protein 1) and *THBS1* (thrombospondin 1). All three genes had significantly upregulated sense transcripts and significantly downregulated antisense transcripts in SLE. The reversed change of sense and antisense transcripts of these genes suggested that the two transcripts are distinctly regulated.

### LncRNA expression

LncRNAs can regulate nearby coding genes as *cis*-regulatory elements. We summarized the correlation of transcription between pairs of lncRNAs and coding genes (Figure S7). When the lncRNAs were located within 100 kb upstream of coding genes, the pairs had a positive correlation on average, regardless of whether the lncRNAs were on the same strand as the coding genes or not. The correlation increased as the distance between lncRNAs and coding genes became closer. On the other hand, when the lncRNAs were located downstream of coding genes, there was no association between the two classes of transcripts if they were on the opposite strands, and a much stronger association if they were on the same strand. The latter might suggest the misclassification of extended 3′ UTR transcription of coding genes as lncRNAs. Although lncRNAs were less likely to change in SLE compared to other RNA classes (Table S4), the locations of some significantly changed lncRNAs suggested their involvement in SLE. For example, both *HIVEP2* itself and a lncRNA about 800 to 1,500 bases upstream of its transcription start site (TSS) were significantly upregulated in SLE, although their RNA abundance was over 50 times different. There were also two significantly upregulated lncRNAs located within 50 kb of each other on chromosome 6 and surrounded by a group of signficantly dysregulated coding genes including *TAGAP*, *FNDC1*, *SOD2*, *WTAP*, and *ACAT2* (Figure S8).

### Pri-miRNA expression

Our RNA purification method did not retain miRNAs, however, pri-miRNAs were easily detected and the combination of increased pri-miRNAs and decreased targets suggested an impact of the miRNAs (Figure S9). Two pri-miRNAs, miR 193a and miR-212, were statistically significantly increased in SLE samples with concomitantly diminished target mRNA levels. MiR-193a, regulating k-ras and survival, was increased by 776% (p = 0.009) [42]. MiR-212, regulating apoptosis, was increased by 148% (p = 0.009) [43]. Thesetwo cellular processes are known to be aberrant in SLE monocytes [44], [45].

We validated mature miR-212-3p differential expression in SLE using qRT-PCR on new samples from six controls and 13 SLE patients (Figure S10). An exogenous control was used because we could not identify an miRNA in monocytes that was unaffected by cytokine treatment (data not shown). The expression of miR-212-5p was too low to be detected in this ligation-based detection strategy. The predicted targets of miR-193a-3p, miR-193a-5p and miR-212 were downregulated by 14.0% to 16.7% in SLE, which was more than the average down-regulation of all other genes (Figure S11).

### Repetitive element expression

Repetitive elements were globally downregulated in SLE (Figure 2C). A closer look at the downregulated repetitive elements showed that they were predominantly ERV classes and were closely correlated with each other, suggesting that they were co-regulated via an upstream mechanism. Interferon is known to repress retroviral activation and over-expression of interferon in SLE has been documented in several studies [1], [2], [46], [47], [48]. Coding genes having the highest positive correlation with the downregulated repetitive elements were potential targets of transcription factors AP1 (p = 3.0E-8), E47 (p = 2.2E-9), RFX1 (p = 3.7E-8), IRF1 (p = 3.5E-2), and IRF2 (p = 4.3E-3), also supporting an effect of interferons.

### Isoform analysis

The proportions of the total reads assigned to each isoform for each gene were compared between the control and SLE samples to evaluate differential isoform transcription. Therefore, this analysis only considered the change of relative isoform abundance while ignoring the gene-level differential expression. This comparison identified 54 genes having one or more isoforms where the relative abundance was significantly changed (p<0.05) by at least 5%, such as *CCR2*, *HIVEP1*, *HIVEP2*, *IL1B*, *IL1R2* and *TLR2*. *SNAPC3* encodes a subunit of a protein complex that activates snRNA. It was previously known as having a single isoform, while our Cufflinks assembly identified a novel splicing site within its 3′UTR (Figure S12). The relative abundance of these two alternative transcripts was changed by about 12% with p = 0.003.

We observed that isoforms where the relative abundance was increased in SLE had significantly more exons than those with decreased relative abundance (Figure S13). This result indicates higher RNA splicing activity and/or more complex RNA processing in SLE monocytes.

We selected two biologically relevant genes to validate the expression of their novel isoforms in monocytes. Primers were designed to selectively bridge the novel exon-exon junction. *IL1R1* and *IRF8* were both found to include a large intragenic exon by RNA-seq (Figures S14 and S15). These exons have no protein coding potential. For IL1R1, the novel exon is an extra 5′ untranslated region. In both cases, PCR demonstrated incorporation of the novel exon (Figure S16).

### Novel loci transcription

**Figure 3:**
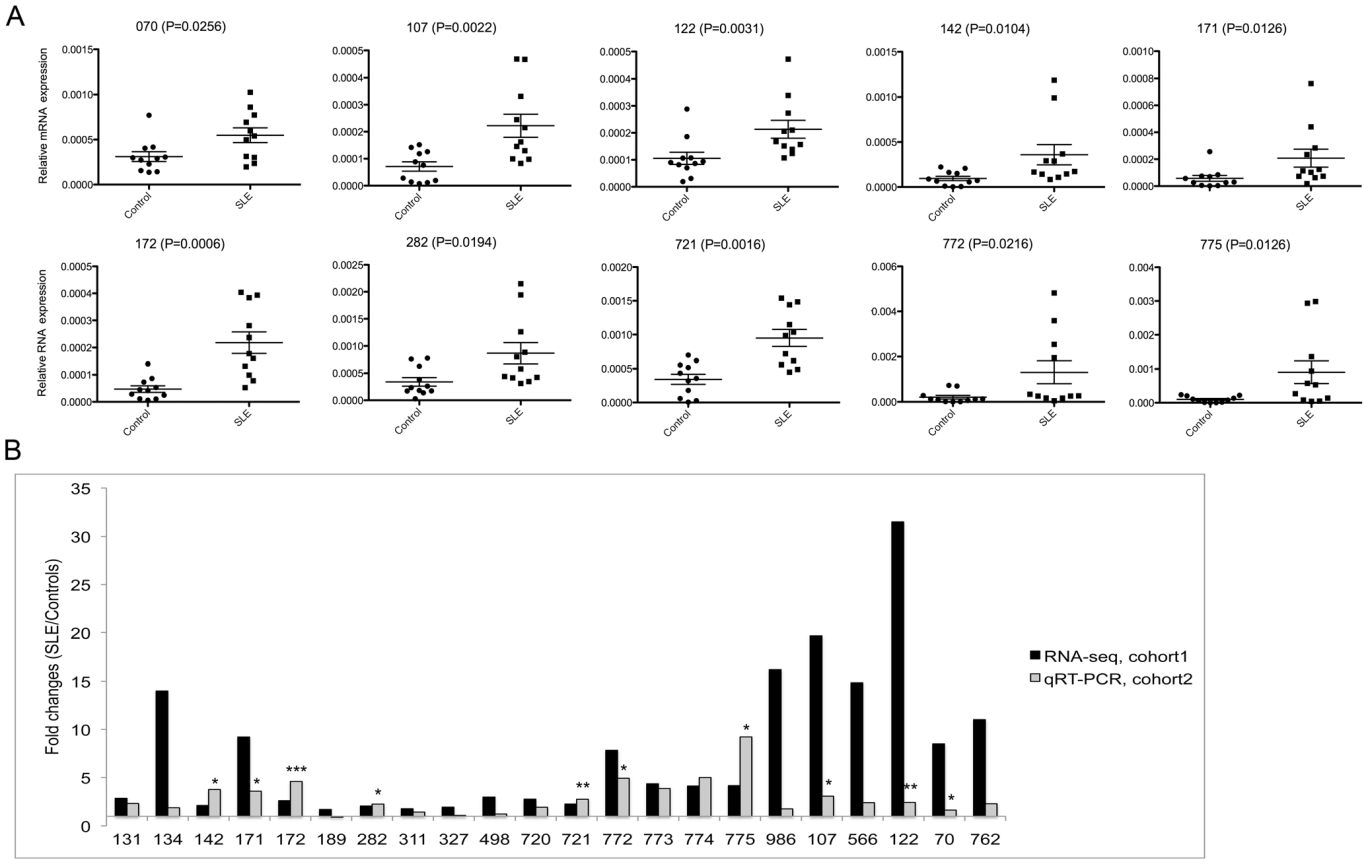
PCR validation in new samples. A) Ten novel loci (70, 107, 122, 142, 171, 172, 282. 721, 772, and 775) were amplified using 11 controls (8 new controls and 3 controls used for the RNA-seq libraries) and 11 new SLE patients. Transcript levels were normalized to β-actin. In each case, the differential expression between SLE and controls was statistically significant with p<0.05, according to the Mann-Whitney test. The cross bars indicate mean and standard error. Primers and locations are given in Methods S1. B) There was a good agreement of expression fold changes in SLE between the RNA-seq and qRT-PCR experiments with p<0.05 (*), p<0.01 (**) or p<0.001(***). All but two of 22 tested novel loci were upregulated in both SLE patient cohorts.

92% differentially expressed novel loci had higher transcription in SLE (Table S4). Many of the novel loci formed clusters located close to each other. For example, three of the four novel loci, located within a 5 kb region on chromosome 18, were among the most significantly upregulated transcripts (Figure S17). Another example was a cluster of 26 novel loci located within a 34 kb region on chromosome 8, most of which were significantly upregulated in SLE and none of which were downregulated (Figure S18). We selected 22 novel transcripts upregulated in SLE to be validated in new samples using qRT-PCR. Ten of the transcripts were significantly upregulated (p = 0.034 to 0.0006) in SLE (Figure 3A), and only two were not upregulated at all (Figure 3B). The other loci were upregulated without reaching statistical significance (Figure 3B and Figure S19).

**Figure 4:**
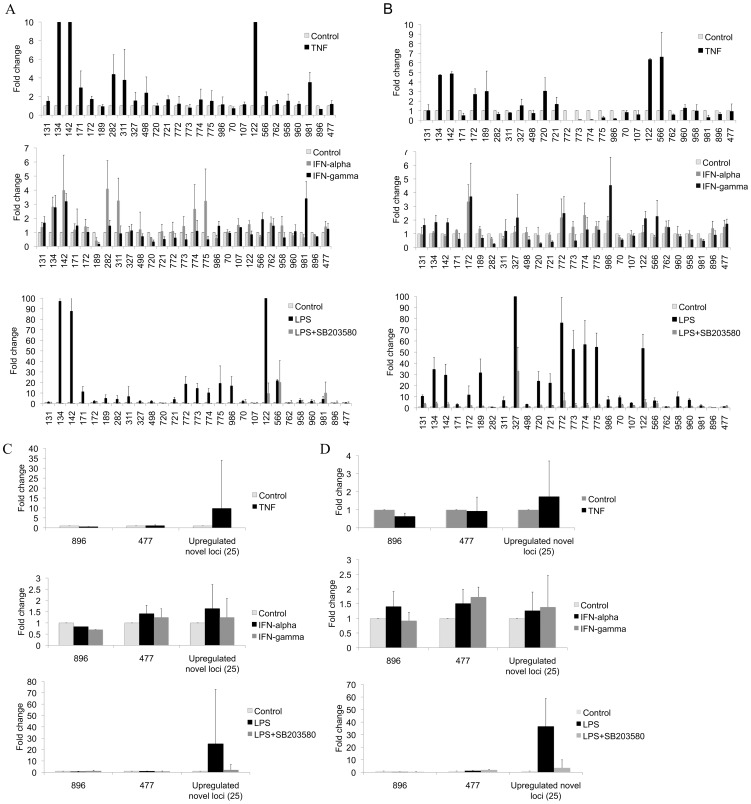
LPS stimulation of monocytes. A) MonoMac 6 cells were stimulated for 16 hours as indicated and qRT-PCR was performed for the novel loci. Only LPS (1 µg/ml) stimulation consistently upregulated these loci. TNF-α (10 ng/ml) led to upregulation of 16/25 (fold change>1.5), αIFN (100 U/ml) led to upregulation of 9/25 (fold change>1.5), γIFN (10 ng/ml) led to upregulation of 5/25 (fold change>1.5), and LPS led to upregulation of 22/25 (fold change>1.5). Three experiments with duplciates or triplicates were averaged. The error bars indicate standard deviation. B) Human primary monocytes were stimulated as above and the abundance of the novel transcripts quantitated by qRT-PCR. TNF-α led to upregulation of 9/25 (fold change>1.5), αIFN led to upregulation of 7/25 (fold change>1.5), γIFN led to upregulation of 9/25 (fold change>1.5), and LPS led to upregulation of 23/25 (fold change>1.5). Pre-treatment of cells with the p38 inhibitor, SB203580 led to markedly diminished induction of expression by LPS. Three experiments with duplciates or triplicates were averaged. The error bars indicate standard deviation. C) The average fold change for the aggregated novel upregulated loci in SLE were calculated for MonoMac 6 cells and D) healthy human primary monocytes stimulated by LPS, TNF-α, αIFN,or γIFN. Locus 896 was down-regulated and locus 477 was not changed in the orignal SLE samples and were included as controls. The error bars indicate standard deviation in C and D.

The fact that these loci were preferentially or exclusively transcribed in SLE monocytes indicates they are very cell-specific. They were not present in seven annotated gene databases supporting SLE-specificity. To examine potential mechanisms driving expression, we initially treated MonoMac 6 cells with TNF-α, α2-interferon, γ–interferon, or LPS. Time course experiments identified the optimal stimulation time. Only LPS replicated the pattern of expression of novel loci seen in the SLE samples (Figure 4A). These findings were further validated using published datasets and examining concordance. In this analysis, genes over-expressed in SLE were equally likely to be represented in the a2-interferon- and LPS-induced genesets (Figure S20). We then confirmed the effect using monocytes from healthy adult donors (Figure 4B). To understand potential pathways regulating the LPS effect, we utilized p38 (SB203580), ERK (U0126) and JNK (SP600125) inhibitors. The major effect of LPS induction of the novel transcripts was p38 mediated (Figures 4C and 4D, Figures S21 and S22).

### Circulating endotoxin

**Figure 5:**
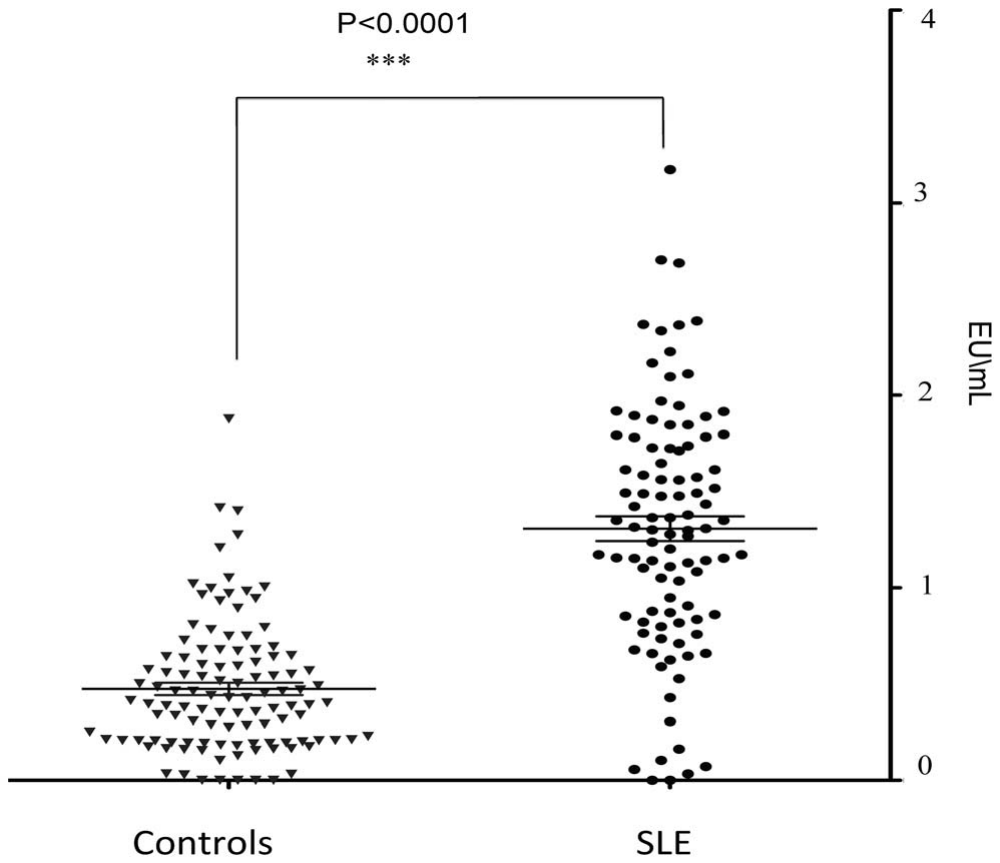
SLE patient circulating endotoxin levels. Circulating endotoxin was quantitated using the Limulus assay. 99 female SLE patients and 112 female Red Cross blood donors were anlayzed. SLE patients had significantly more endotoxin on average than controls (P<0.0001). The cross bars indicate the mean and standard error.

Although endotoxin has been previously implicated in murine lupus models and LPS is known to activate interferon pathways via TLR4, there has been no direct measurement of endotoxin in SLE patients [16], [17], [18], [19], [20], [21]. We compared, therefore, serum levels from 99 female SLE patients and those from 112 female healthy adult blood donors. This patient set comprised new samples. The SLE patients were 91% female, 46% black, 54% Caucasian, mean age 37.1 years, mean SLEDAI (Systemic Lupus Erythematosus Disease Activity Index) of 2.6 and mean physician global estimate of 0.45. The mean prednisone dose was 9 mg/day. The red cross blood donors were females who were self declared as healthy. SLE patients had significantly higher endotoxin levels compared to controls (Figure 5). When we examined clinical markers for associations with circulating endotoxin levels, we found no association with age, weight, erythrocyte sedimentation rate (ESR), C-reactive protein (CRP), specific organ damage, specific autoantibodies or complete blood count (CBC) parameters. Because LPS/endotoxin can induce type I interferon expression, we examined the concordance of coding genes expressed in SLE, after stimulation with LPS and after stimulation with α-interferon using our prior results and published array results [49], [50]. There was substantial overlap, demonstrating that endotoxin can in part mimic the type I interferon signature seen in SLE (Figure S21).

## Discussion

With the advent of next-generation sequencing technologies a more comprehensive and accurate transcriptional analysis has become feasible. We report a whole transcriptome analysis of patients with SLE and compare gene expression with that of healthy controls. We detected many instances of SLE-specific alternative splicing, alternative polyadenylation, and novel loci transcription. Splicing and polyadenylation in SLE both favored longer, more complex transcripts.

One of our goals was to identify the transcript abundance of non-coding RNAs, which have been demonstrated in subjects with Aicardi Goutières syndrome, an infantile-onset disorder with features of lupus, and in murine models, to drive a type I interferon signature [51], [52], [53]. In this study, we found instead decreased expression of many non-coding RNAs. We hypothesize that repression of endogenous retroviral sequences may be mediated by the type I interferon that is known to be overexpressed in SLE patients [54]. One class of non-coding RNA for which expression was clearly induced in SLE patients was that of the pri-miRNAs. These small non-coding RNAs are processed to repress translation and regulate multiple messenger RNAs. In our study, two specific pri-miRNAs were significantly upregulated in SLE monocytes compared to healthy controls. In addition, we were able to demonstrate that the targets of these two miRNAs exhibited decreased message levels. These observations suggest that the elevated pri-miRNA levels that we identified in SLE monocytes are functionally relevant.

Among known protein-coding genes, there was evidence of global repression, and many genes downregulated in SLE monocytes were related to cell proliferation and cell adhesion. The genes upregulated in SLE monocytes reflected active inflammation. These observations are concordant with what has been seen using arrays and what is known about monocyte behavior in SLE [1], [2], [25], [55], [56], [57], [58], [59]. Monocytes are known to have a shortened life span and to exhibit characteristics related to type I interferon exposure [1], [23], [45]. The overall repression of gene expression could be consistent with increased differentiation and our previous studies demonstrated altered surface expression of several proteins [14].

The finding of SLE-specific isoforms and polyadenylation was particularly intriguing. The finding of disease-specific isoforms is not unique to this study, but it has not been reported this extensively outside of tumor-specific transcripts. Some studies have found functional effects of autoantibodies directed at nuclear constituents and altered expression of splicing factors and this study raises the question of the mechanism driving altered processing [60], [61], [62]. Altered expression of splicing factors could also contribute to the pattern observed here [61], [62].

This study raises many questions. We examined a cohort of patients with low disease activity to minimize the effect of medications. Whether patients with more severe disease could have a more disturbed transcriptome is not known. This is a relatively small cohort size, and additional studies will be required to replicate these findings. We did not perform extensive validation of the many classes of RNA found to have altered expression, instead capturing a snapshot of the SLE transcriptome in a single cell type and focusing on the breadth of the effect. We validated using new samples from controls and patients for the DEGs and found similar changes in nearly all DEGs, supporting that these effects are consistently seen in the disease. If similar disruptions are found in additional cell types, it would suggest a systematically altered transcriptome. Nevertheless, analyses of isoforms and un-annotated loci is a moving target and additional information is likely to be forthcoming, allowing improved understanding of the regulation of these processes. Monocytes represent a cell type that is uniquely plastic and it may be that effects are magnified in this cell type.

IRF1, IRF2, and RFX1 were previously identified by us as potential regulators of genes with altered histone H4 acetylation [23], [24], [25]. These transcription factors, now identified as potential regulators of the SLE transcriptome, could integrate inflammatory and interferon signals. The MAP kinase and NFκB pathways were identified as potential regulating pathways in the polyadenylation pattern specific to the SLE samples. These pathways have long been implicated in SLE [63], [64], [65], [66], [67], [68]. To further implicate the MAP kinase pathway, we found that expression of many novel loci were inducible with LPS and that inducibility could be blocked with the P38 inhibitor, SB203580. The roles of the novel loci are not yet known and we acknowledge that these fall into a bioinformatic limbo. Nevertheless, endotoxin has been implicated in a variety of diseases and induces type I IFN [69], [70], [71], [72], [73], [74], [75], [76]. Our findings of significant overlap of the genes induced by LPS/endotoxin, SLE and α-interferon along with increased endotoxin in peripheral blood supports a role for endotoxin in the pathologic gene expression pattern identified here. This is the first RNA-seq study of SLE monocytes and our analysis revealed a surprisingly distorted transcriptome. The strengths of the study include the comprehensive approach to the characterization of the transcriptional landscape, use of a purified cell type, validation of the findings using a new cohort, and a mechanistic insight into the expression of novel loci.

Limitations of this study include a small sample size focused on mild to moderate disease activity. While the low disease activity enabled us to examine patient samples without the perturbation of high-level immune suppression, it may also have limited our findings. The platform used and the sample cell count may also have limited our findings. The libraries were 50 bp single reads and total RNA was used with post-run ribosomal RNA exclusion. Finally, technical aspects such as RNA quality may have limited our ability to identify disease-specific variation in signal. Nevertheless, in spite of these potential limitations, our analyses were robust and identified many changes specific to the SLE transcriptome.

In summary, we found a broadly altered transcriptome. By using a single cell type, we minimized the effects of different cell populations and improved the specificity of our discoveries. The most significant finding of this study was identification of disease-specific novel loci expression, regulated by endotoxin. Circulating endotoxin has been negatively associated with prognosis in a number of diseases and is thought to drive a type of immune exhaustion [77], [78]. Whether endotoxin could be responsible for other features of the altered transcriptome or could represent a biomarker for disease severity remains to be determined. While additional studies will be required to determine which features contribute to the pathologic processes in this still enigmatic disease, the importance of this study lies in the identification of multiple features of altered transcription and processing in SLE, a heretofore unappreciated facet of the disease.

## Supporting Information

Figure S1 **Overall transcriptome characteristics.** A) The Tophat-Cufflinks pipeline identified four major classes of transcripts from 17 RNA-seq libraries. RefSeq genes constituted the majority of the transcripts. B) Novel isoforms and loci were different from known transcripts in terms of average length and numbers of exons on average. C) Coding RNA was the most abundant RNA species (except ribosomal RNA) in monocytes based on the count of RNA-seq reads. Non-coding RNA collectively accounted for approximately 20% of total RNA. D) Small RNAs had the highest expression level on average after adjusting read counts for the total length of RNA classes.
(DOCX)

Figure S2 **High novelty and high confidence novel isoforms and loci.** A) Novel isoforms including at least one unknown exon-exon junction or mapped by ≥10 unique reads in at least six libraries were considering as having high-novelty or high-confidence, respectively. B) Novel loci not overlapping any known transcribed region or mapped by ≥10 unique reads in at least six libraries were considering as having high-novelty or high-confidence, respectively. Odds ratios and p values were the result of Fisher's Exact test performed on the overlap.
(PDF)

Figure S3 **Validation of novel transcripts.** Transcripts were validated for 27 identified novel loci using qRT-PCR (black bars). Primary monocyte RNA was used as the source and control amplifications using non-reverse-transcribed RNA were used as the negative control (No RT bars). Globin, not expected to be expressed in monocytes, was used as an additional negative control. Beta-actin was used for normalization. Most of these novel loci were generally expressed at low levels. The locations of the novel loci are in Methods S1. This represents n = 1. Further validation appears in the main text.
(DOCX)

Figure S4 **Validation of differential gene expression.** The differential expression of six coding genes in SLE were validated by qRT-PCR. The samples consisted of 11 controls (including 3 internal validation samples from which the RNA-seq libraries were made) and 11 new SLE patients. Five of the genes were validated as having significant change in SLE. The sixth gene, *CD177*, had the same direction of change in SLE samples but the change did not reach statistical significance. The cross bars indicate mean and standard error according to the Mann-Whitney test.
(DOCX)

Figure S5 **3′ UTR length networks.** A) The genes with longer 3′UTRs in SLE patients were networked using Ingenuity. The two most dominant networks are shown. NFκB, AKT, and UBC are the dominant nodes. B) The genes with shorter 3′UTRs in SLE patients were networked using Ingenuity. The two most dominant networks are shown. MAP kinases and UBC were the dominant nodes. Data output from Ingenuity is shown in the Tables below.
(DOCX)

Figure S6 **Sense- antisense expression.** About 5,000 coding genes had detectable antisense transcription in monocytes. A) The average transcription of antisense transcripts was less than the half of the corresponding sense transcripts; but a small number of antisense transcripts had over ten-fold higher transcription of their sense counterparts. B) The transcription of sense and antisense pairs tended to be positively correlated. The correlation between each pair was first calculated across samples in the control and SLE groups separately, then the two correlation coefficients were combined using Fisher's transformation.
(DOCX)

Figure S7 **LncRNA association with adjacent transcription.** The co-regulation of lncRNAs and their nearby coding genes was dependent on their distance and relative location. The horizontal black line is indicates the average correlation of random pairs of lncRNAs and coding genes. (Oppo: opposite strand.)
(DOCX)

Figure S8 **Chromosome 6 lncRNA cluster.** A cluster of coding genes and lncRNAs located on chr6q25.3 were commonly dysregulated in SLE monocytes. Four coding genes with medium to high transcription levels were all upregulated in SLE while the other coding gene, *FNDC1*, had a very low transcription level and was downregulated in SLE. Both highlighted lncRNAs were significantly upregulated in SLE. Three other lncRNAs within this region had detectable transcription, but no significant changes in SLE. The Table demonstrates the read counts for each locus.
(DOCX)

Figure S9 **MicroRNA- target correlation.** Ten pri-miRNAs and their predicted targets had a negative correlation in monocytes. The correlation was calculated using normalized read counts of pri-miRNAs and target coding genes. The correlation coefficients of each miRNA-target pair were obtained from the control and SLE groups separately and then combined using Fisher's transformation. Each bar represents the average and standard error of correlation coefficients of a target list. Target lists were downloaded from miRBase.
(DOCX)

Figure S10 **miRNA validation.** The increased expression of miR-212-3p in SLE was validated by qRT-PCR with cel-miR-238 as a control. Six new controls and 13 new SLE samples were used.
(DOCX)

Figure S11 **High expression miRNA target transcript expression.** Three miRNAs whose pri-miRNAs had higher expression in SLE were examined for an effect on potential target transcripts. For these three miRNAs, target transcript expression was significantly downregulated on average. While coding genes were generally downregulated in SLE, the target genes tended to be downregulated even more. Target lists were downloaded from miRBase database.
(DOCX)

Figure S12 **Isoform distribution for** ***SNAPC3*** **.** A) Cufflinks assembly based on our RNA-seq data identified a novel splicing site within the 3′ UTR of *SNAPC3*. B) The relative abundance of the two isoforms was changed in SLE. C) The difference in relative abundance was statistically significant.
(DOCX)

Figure S13 **Isoforms with increased expression in SLE are more complex.** The isoforms where the relative abundance was increased in SLE had a higher number of exons than those where the relative abundance was decreased (p = 0.007). On average, the SLE-favored isoforms had about 0.5 exons more than the control-favored isoforms.
(DOCX)

Figure S14 **Novel isoforms of IL1R1.** Tophat-Cufflinks identified three isoforms of *IL1R1* not included in the RefSeq annotation. Two of them have been included in GENCODE database version 14 (**A&B**), and both had histone patterns at their transcription start sites consistent with expression according to ENCODE histone modification data sets generated from CD14+ monocytes (**C&D**). The other isoform, *TCONS_00030117*, had ∼6 kb extra 5′ UTR exon (**E**). According to the ENCODE data, this exon has a strong H3K9me3 footprint (**F**), which is known as a repressive histone modification, suggesting unique transcriptional regulation at this region.
(DOCX)

Figure S15 **Novel isoform of IRF8.** Tophat-Cufflinks identified a novel isoform of *IRF8*, which included a prolonged exon 4. The existence of this isoform was supported by an ENCODE RNA-seq data set generated from 9 cell lines, including lymphoblastoid cell line GM12878. Transcription was detected in GM12878 cross the full gene body of IRF8, but the extended region of exon 4 in the novel isoform had higher transcription level than those of the introns. This isoform is likely an intermediate product of RNA processing and not present in the mature mRNA.
(DOCX)

Figure S16 **Novel isoform validation.** A) Tophat-Cufflinks identified multiple novel isoforms of *IL1R1*, each with a new exon-exon junction in the 5′ UTR. One of the isoforms (in red) was validated by qRT-PCR using a pair of primers across two exons (F4/R4). B) *IRF8* was known to have a single isoform. Tophat-Cufflinks identified a novel isoform, which was validated by qRT-PCR. These gels are representative of three experiments, with comparable results.
(DOCX)

Figure S17 **Novel transcripts located on Chromosome 18.** The detailed annotation of a cluster of novel transcripts located on chromosome 18 using public genomic data. A) Four novel transcripts identified by the Tophat-Cufflinks pipeline in monocytes about 20 kb upstream of coding gene *SERPINB2* are shown. All four transcripts were transcribed at the opposite direction as *SERPINB2*, and three of them were significantly upregulated in SLE by 303% to 671% (p = 1.3E-6 to 1.5E-8). All four transcripts were validated by qPCR. B) These transcripts are not included in commonly used gene annotation databases. Two predicted genes partially overlap with these transcripts, but both are transcribed in the opposite direction. C) Sequences within this region are evolutionarily conserved. D) A very low level of transcription was detected within this region according to an ENCODE RNA-seq data set that measured transcriptomes in nine cell lines, not including monocytes. E) This region has a DNase I hypersensitive site in CD14+ monocytes according to another ENCODE data set. F) Furthermore, an ENCODE data set measuring various histone marks in CD14+ monocytes showed that this region has a nucleosome pattern indicative of active transcription.
(DOCX)

Figure S18 **Novel transcripts located on Chromosome 8.** A cluster of 26 novel transcripts located on chromosome 8 within a ∼34 kb region upstream of coding gene *ADAM28*. A) All of the loci are transcribed in the opposite direction of *ADAM28*. B) None of them are included in a common gene annotation databases. Fifteen of these transcripts were significantly upregulated in SLE (p<0.01) by 80% to 3435% and none of them were downregulated in SLE. C) Most of the transcripts are located within evolutionarily conserved regions. D) ENCODE data detected very low levels of transcripts within this region in 9 cell lines. E) According to ENCODE data sets generated from monocytes, there is a DNase I hypersensitivity site within this region. F) The same sites showed a histone modification pattern of active nucleosomes.
(DOCX)

Figure S19 **Differential expression of novel loci.** Twelve novel loci were amplified using 11 controls (including three internal validation controls used for the RNA-seq libraries and eight new controls) and 11 new SLE samples using qRT-PCR. All genes were normalized to β-actin. P values according to Mann-Whitney in each case are given in parentheses. Only two failed to demonstrate increased expression in this new SLE cohort. Locations are given in Table S3. The cross bars indicate mean and standard error.
(DOCX)

Figure S20 **Concordance of LPS, interferon and SLE gene expression.** Five LPS data sets were created from four GEO monocyte data series (additional information on methods available in Methods S1). The α-interferon data set is from our previously published work. The coding genes shown to be upregulated in SLE were analyzed for upregulation after LPS stimulation and α-interferon (aIFN) treatment. A) All three LPS data sets demonstrated that LPS treatment also increased expression of the genes shown to be upregulated in SLE. N = the number of unique genes included in the analysis. B) The degree of overlap between SLE-induced genes and aIFN-induced genes was comparable to the degree of overlap between SLE-induced genes and LPS-induced genes.
(DOCX)

Figure S21 **The effect of different MAP kinase inhibitors on LPS-induced novel loci expression.** MonoMac6 cells (above) and primary monocytes (below) were treated with p38 (SB203580), ERK (U0126) and JNK (SP600125) inhibitors for 30 minutes and then stimulated with LPS. The y-axis represents the ratio of the LPS+ inhibitor-treated cells over the LPS alone treated cells, with the horizontal line indicating equivalence. The p38 inhibitor led to most diminished expression of the novel loci. At the right of the graph, loci 477 and 896 were included as controls, which were novel loci that were not altered in SLE. N = 3.
(DOCX)

Figure S22 **The average of the novel loci expression.** The expression levels of the novel loci, demonstrated in Figure S21, were averaged to better highlight the effect of the inhibitors. p38 (SB203580), ERK (U0126) and JNK (SP600125) inhibitors were used to define the role of this pathway on the novel loci expression. A) MonoMac6 cell results from Figure S21 were averaged to demonstrate the overall effect of the MAP kinase inhibitors. B) Similarly, in primary monocytes, the results from Figure S21 were averaged. TNF was used as a positive control for the LPS stimulation in C) MonoMac6 cells and D) primary monocytes.
(DOCX)

Table S1 **Clinical characteristics of SLE patients.**
(DOCX)

Table S2 **RNA Samples for Libraries.**
(DOCX)

Table S3 **Locations of novel loci.**
(DOCX)

Table S4 **Number of differentially expressed genes/transcripts in each class.**
(DOCX)

Table S5 **Functional categorization of differentially expressed gene by DAVID.**
(DOCX)

Table S6 **Cross-referencing of genes identified in genetic association studies and significantly upregulated in this study.**
(DOCX)

Data S1 **Processed Data.**
(XLS)

Methods S1 **Supplemental Methods List.**
(DOCX)

References S1 **Supplemental References List: Tables.**
(DOCX)
