# Supplementary figures and images for: The SLE Transcriptome Exhibits Evidence of Chronic Endotoxin Exposure and Has Widespread Dysregulation of Non-Coding and Coding RNAs
Source: PLoS One. 2014 May 5;9(5):e93846. doi: 10.1371/journal.pone.0093846 (PMC4010412; doi:10.1371/journal.pone.0093846)

**Figure S1. Overall transcriptome characteristics**


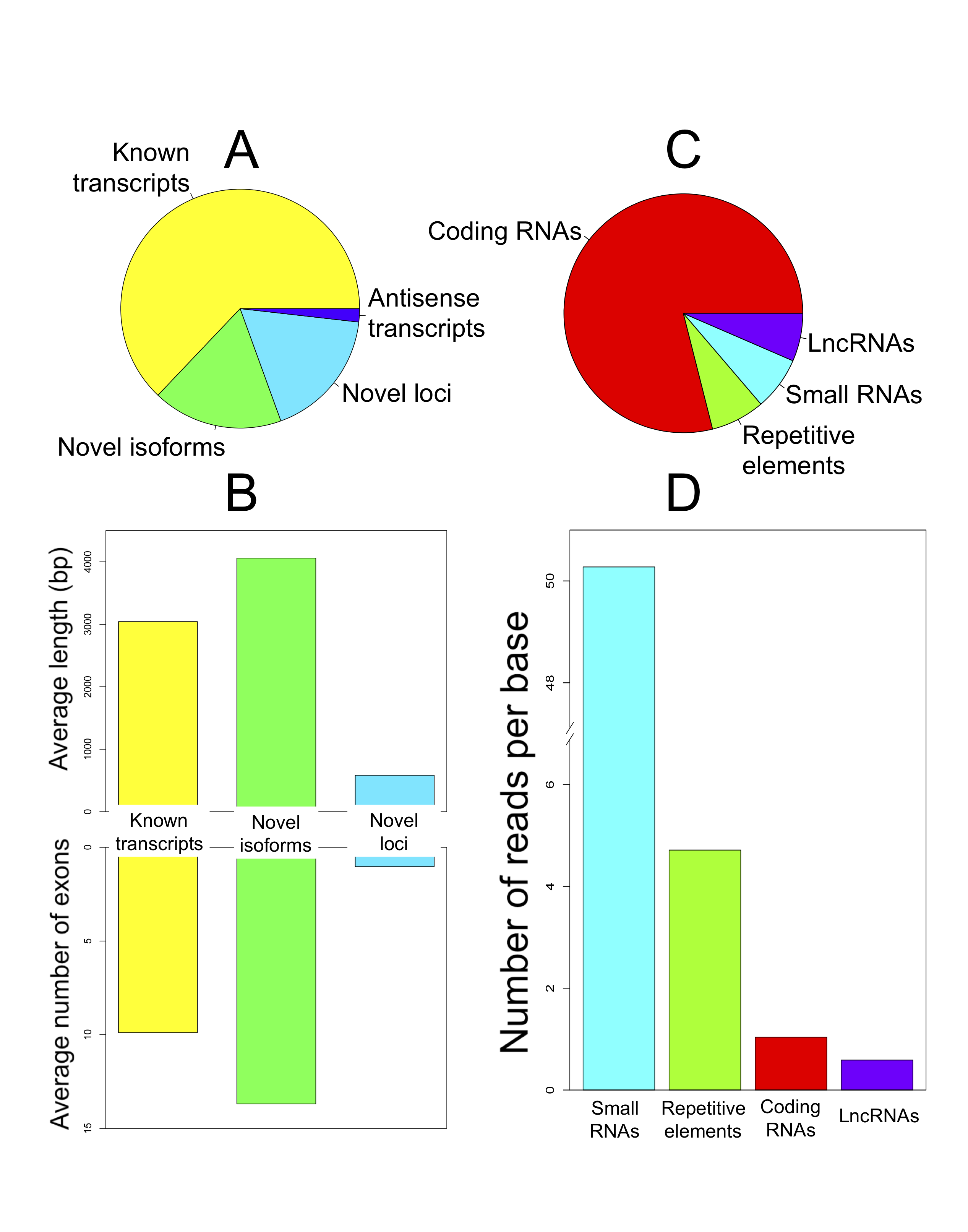

Supplement: Figure S1 — Overall transcriptome characteristics. A) The Tophat-Cufflinks pipeline identified four major classes of transcripts from 17 RNA-seq libraries. RefSeq genes constituted the majority of the transcripts. B) Novel isoforms and loci were different from known transcripts in terms of average length and numbers of exons on average. C) Coding RNA was the most abundant RNA species (except ribosomal RNA) in monocytes based on the count of RNA-seq reads. Non-coding RNA collectively accounted for approximately 20% of total RNA. D) Small RNAs had the highest expression level on average after adjusting read counts for the total length of RNA classes. (DOCX) [file pone.0093846.s001.docx]

**Figure S2. High novelty and high confidence novel isoforms and loci**

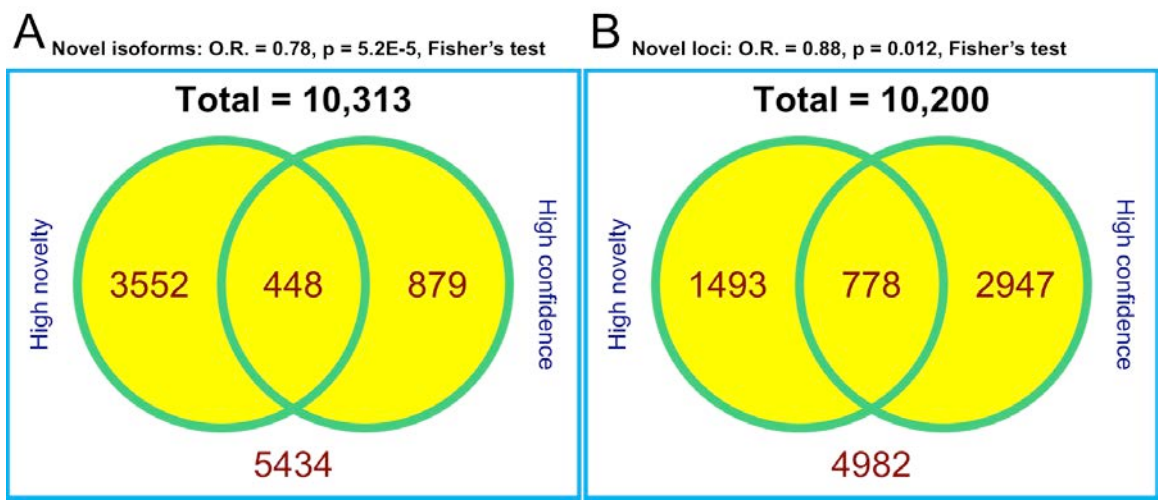

Supplement: Figure S2 — High novelty and high confidence novel isoforms and loci. A) Novel isoforms including at least one unknown exon-exon junction or mapped by ≥10 unique reads in at least six libraries were considering as having high-novelty or high-confidence, respectively. B) Novel loci not overlapping any known transcribed region or mapped by ≥10 unique reads in at least six libraries were considering as having high-novelty or high-confidence, respectively. Odds ratios and p values were the result of Fisher's Exact test performed on the overlap. (PDF) [file pone.0093846.s002.pdf]

**Figure S4. Validation of differential gene expression**


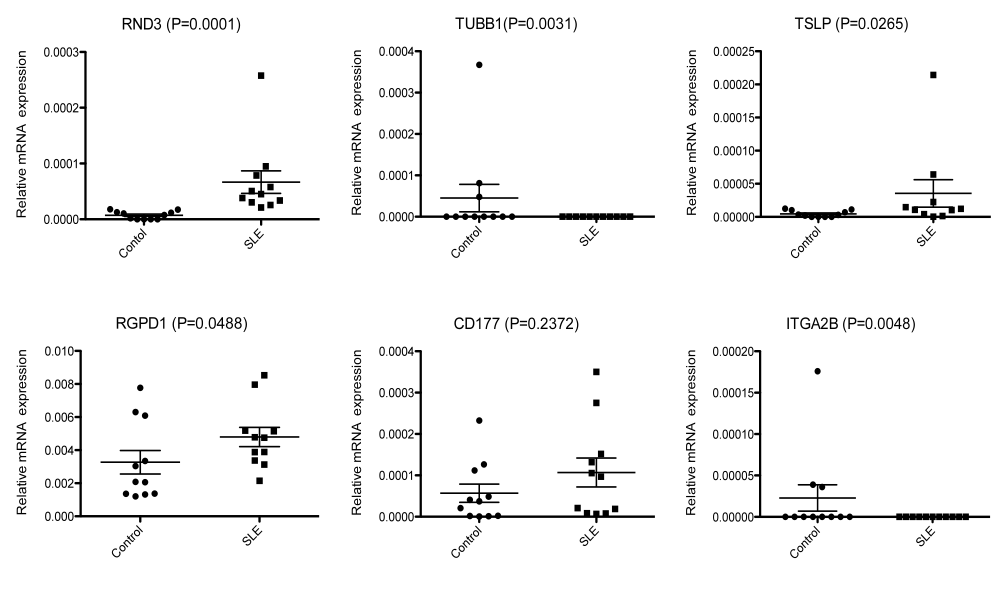

Supplement: Figure S4 — Validation of differential gene expression. The differential expression of six coding genes in SLE were validated by qRT-PCR. The samples consisted of 11 controls (including 3 internal validation samples from which the RNA-seq libraries were made) and 11 new SLE patients. Five of the genes were validated as having significant change in SLE. The sixth gene, CD177, had the same direction of change in SLE samples but the change did not reach statistical significance. The cross bars indicate mean and standard error according to the Mann-Whitney test. (DOCX) [file pone.0093846.s004.docx]

**Figure S8. Chromosome 6 lncRNA cluster**


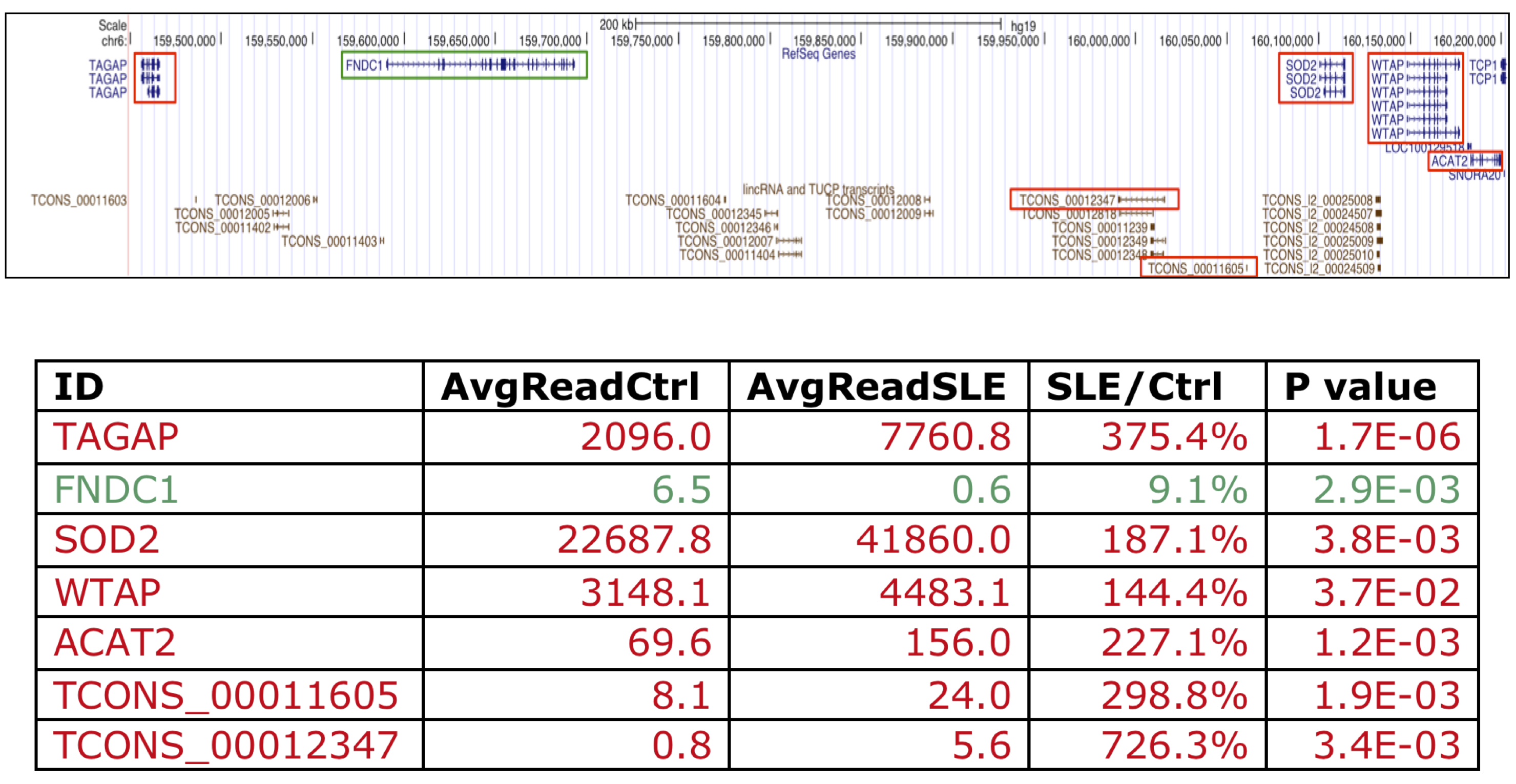

Supplement: Figure S8 — Chromosome 6 lncRNA cluster. A cluster of coding genes and lncRNAs located on chr6q25.3 were commonly dysregulated in SLE monocytes. Four coding genes with medium to high transcription levels were all upregulated in SLE while the other coding gene, FNDC1, had a very low transcription level and was downregulated in SLE. Both highlighted lncRNAs were significantly upregulated in SLE. Three other lncRNAs within this region had detectable transcription, but no significant changes in SLE. The Table demonstrates the read counts for each locus. (DOCX) [file pone.0093846.s008.docx]

**Figure S12. Isoform distribution for *SNAPC3***


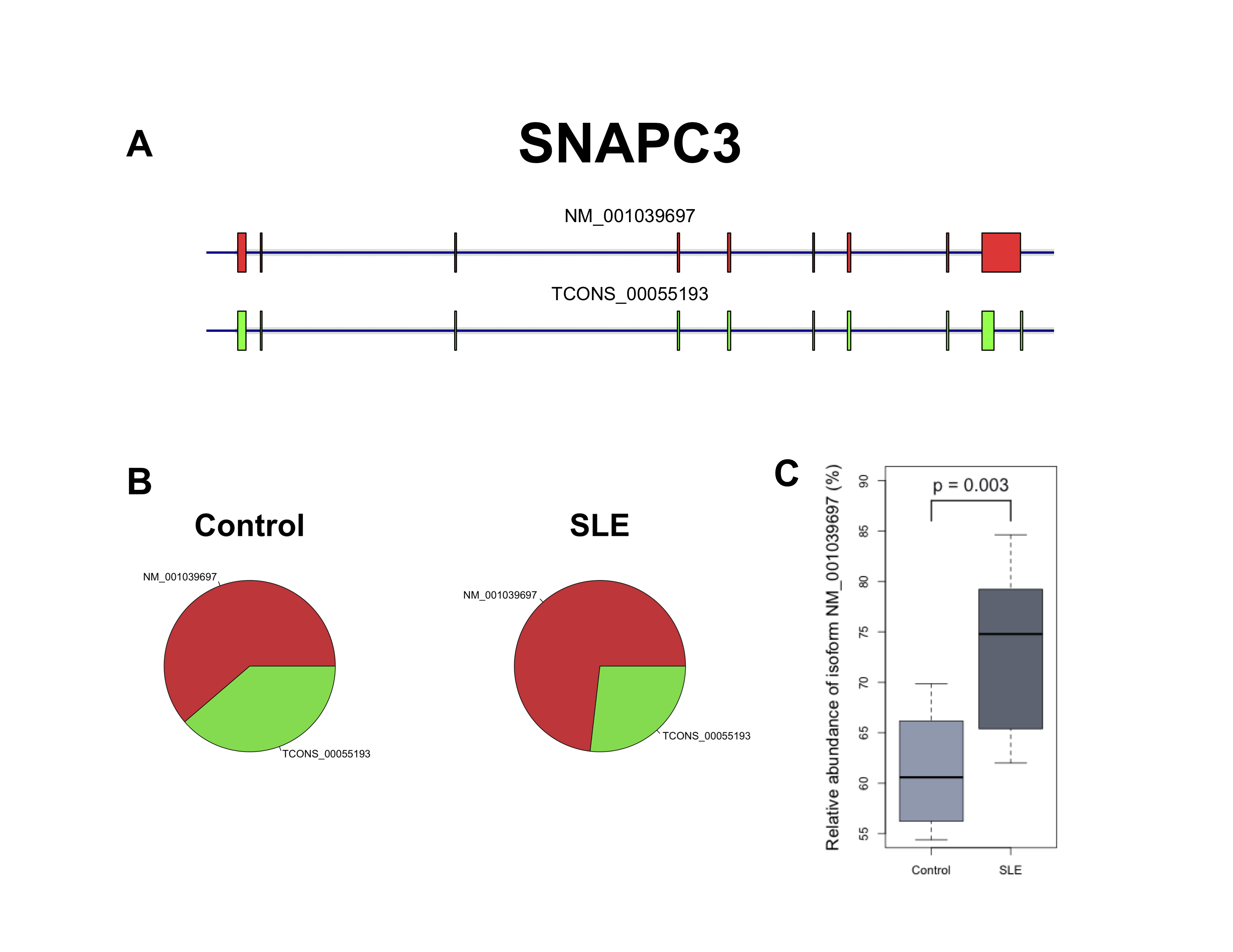

Supplement: Figure S12 — Isoform distribution for SNAPC3 . A) Cufflinks assembly based on our RNA-seq data identified a novel splicing site within the 3′ UTR of SNAPC3. B) The relative abundance of the two isoforms was changed in SLE. C) The difference in relative abundance was statistically significant. (DOCX) [file pone.0093846.s012.docx]

**Figure S14. Novel isoforms of IL1R1**


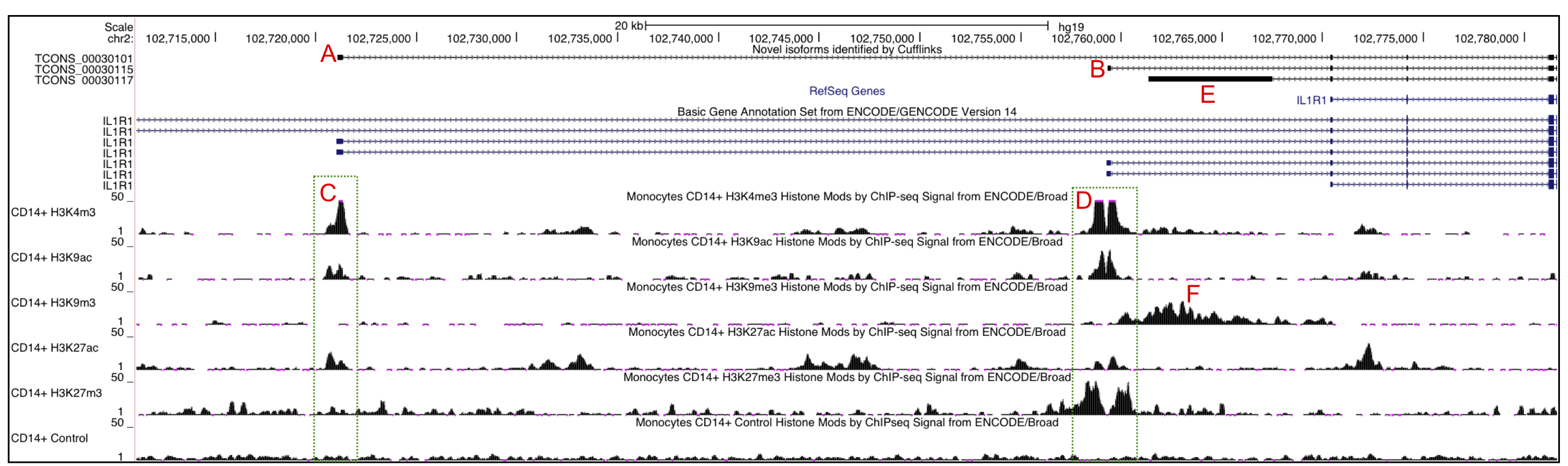

Supplement: Figure S14 — Novel isoforms of IL1R1. Tophat-Cufflinks identified three isoforms of IL1R1 not included in the RefSeq annotation. Two of them have been included in GENCODE database version 14 (A&B), and both had histone patterns at their transcription start sites consistent with expression according to ENCODE histone modification data sets generated from CD14+ monocytes (C&D). The other isoform, TCONS_00030117, had ∼6 kb extra 5′ UTR exon (E). According to the ENCODE data, this exon has a strong H3K9me3 footprint (F), which is known as a repressive histone modification, suggesting unique transcriptional regulation at this region. (DOCX) [file pone.0093846.s014.docx]

**Figure S15. Novel isoform of IRF8**


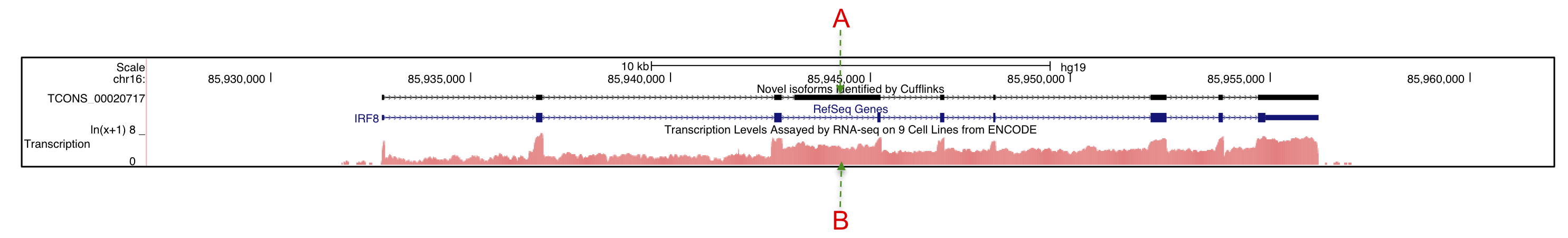

Supplement: Figure S15 — Novel isoform of IRF8. Tophat-Cufflinks identified a novel isoform of IRF8, which included a prolonged exon 4. The existence of this isoform was supported by an ENCODE RNA-seq data set generated from 9 cell lines, including lymphoblastoid cell line GM12878. Transcription was detected in GM12878 cross the full gene body of IRF8, but the extended region of exon 4 in the novel isoform had higher transcription level than those of the introns. This isoform is likely an intermediate product of RNA processing and not present in the mature mRNA. (DOCX) [file pone.0093846.s015.docx]

**Figure S16. Novel isoform validation**


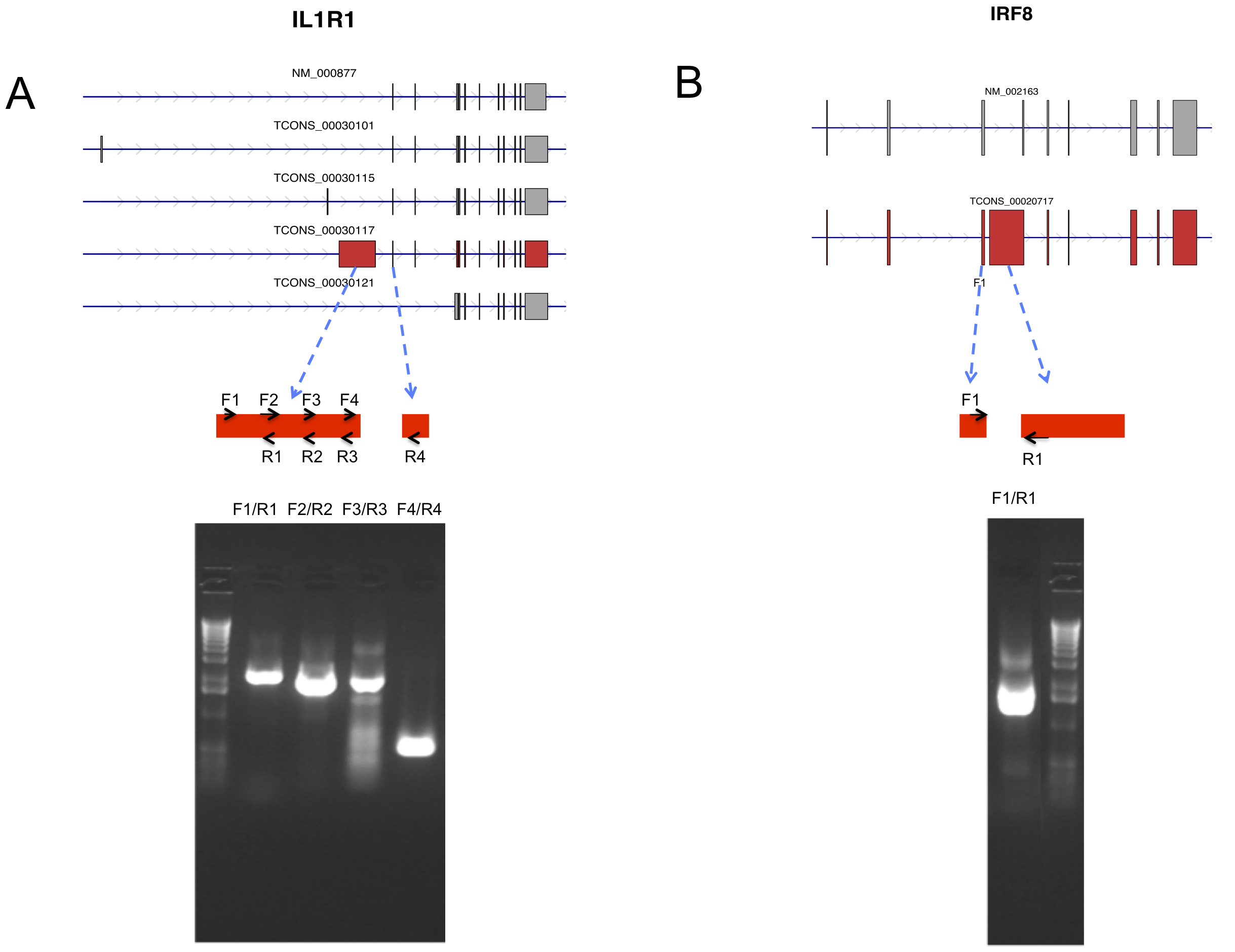

Supplement: Figure S16 — Novel isoform validation. A) Tophat-Cufflinks identified multiple novel isoforms of IL1R1, each with a new exon-exon junction in the 5′ UTR. One of the isoforms (in red) was validated by qRT-PCR using a pair of primers across two exons (F4/R4). B) IRF8 was known to have a single isoform. Tophat-Cufflinks identified a novel isoform, which was validated by qRT-PCR. These gels are representative of three experiments, with comparable results. (DOCX) [file pone.0093846.s016.docx]

**Figure S19. Differential expression of novel loci**

**
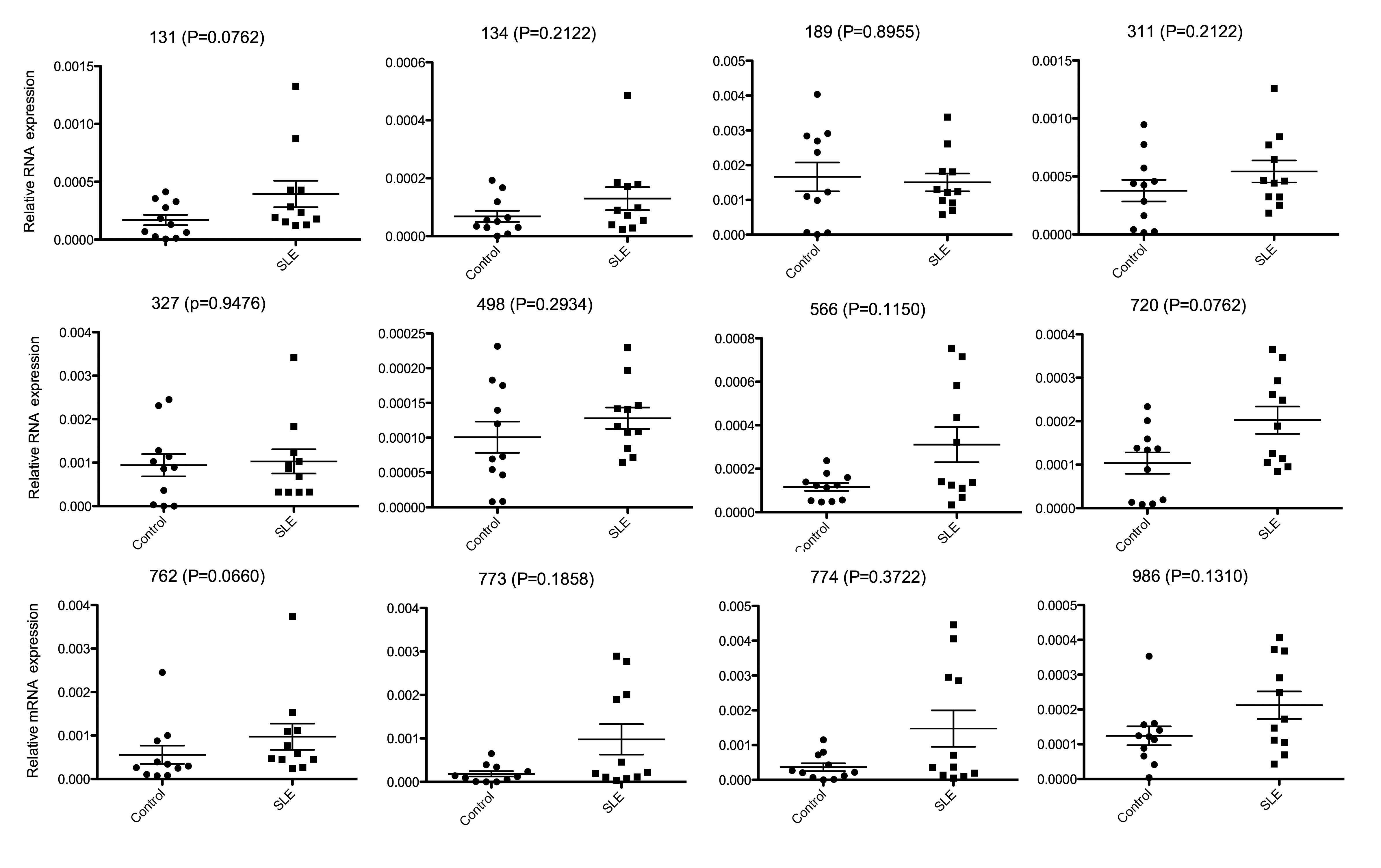
**

Supplement: Figure S19 — Differential expression of novel loci. Twelve novel loci were amplified using 11 controls (including three internal validation controls used for the RNA-seq libraries and eight new controls) and 11 new SLE samples using qRT-PCR. All genes were normalized to β-actin. P values according to Mann-Whitney in each case are given in parentheses. Only two failed to demonstrate increased expression in this new SLE cohort. Locations are given in Table S3. The cross bars indicate mean and standard error. (DOCX) [file pone.0093846.s019.docx]
