# Supplementary material for: The SLE Transcriptome Exhibits Evidence of Chronic Endotoxin Exposure and Has Widespread Dysregulation of Non-Coding and Coding RNAs
Source: PLoS One. 2014 May 5;9(5):e93846. doi: 10.1371/journal.pone.0093846 (PMC4010412; doi:10.1371/journal.pone.0093846)
Supplement: Figure S3 — Validation of novel transcripts. Transcripts were validated for 27 identified novel loci using qRT-PCR (black bars). Primary monocyte RNA was used as the source and control amplifications using non-reverse-transcribed RNA were used as the negative control (No RT bars). Globin, not expected to be expressed in monocytes, was used as an additional negative control. Beta-actin was used for normalization. Most of these novel loci were generally expressed at low levels. The locations of the novel loci are in Methods S1. This represents n = 1. Further validation appears in the main text. (DOCX) [file pone.0093846.s003.docx]

**Figure S3. Validation of novel transcripts**
