# Supplementary material for: The SLE Transcriptome Exhibits Evidence of Chronic Endotoxin Exposure and Has Widespread Dysregulation of Non-Coding and Coding RNAs
Source: PLoS One. 2014 May 5;9(5):e93846. doi: 10.1371/journal.pone.0093846 (PMC4010412; doi:10.1371/journal.pone.0093846)
Supplement: Figure S5 — 3′ UTR length networks. A) The genes with longer 3′UTRs in SLE patients were networked using Ingenuity. The two most dominant networks are shown. NFκB, AKT, and UBC are the dominant nodes. B) The genes with shorter 3′UTRs in SLE patients were networked using Ingenuity. The two most dominant networks are shown. MAP kinases and UBC were the dominant nodes. Data output from Ingenuity is shown in the Tables below. (DOCX) [file pone.0093846.s005.docx]

**Figure S5. 3’ UTR length networks**

| **Long UTR Pathway Analysis** | | |
| --- | --- | --- |
| **Category** | **P value** | **# molecules** |
| Cancer | 1.5 X 10^-4^ | 29 |
| Antimicrobial Response | 3.9 X 10^-5^ | 3 |
| Cell Morphology | 2.2 X 10^-5^ | 13 |
| Cellular Assembly | 2.3 X 10^-4^ | 9 |


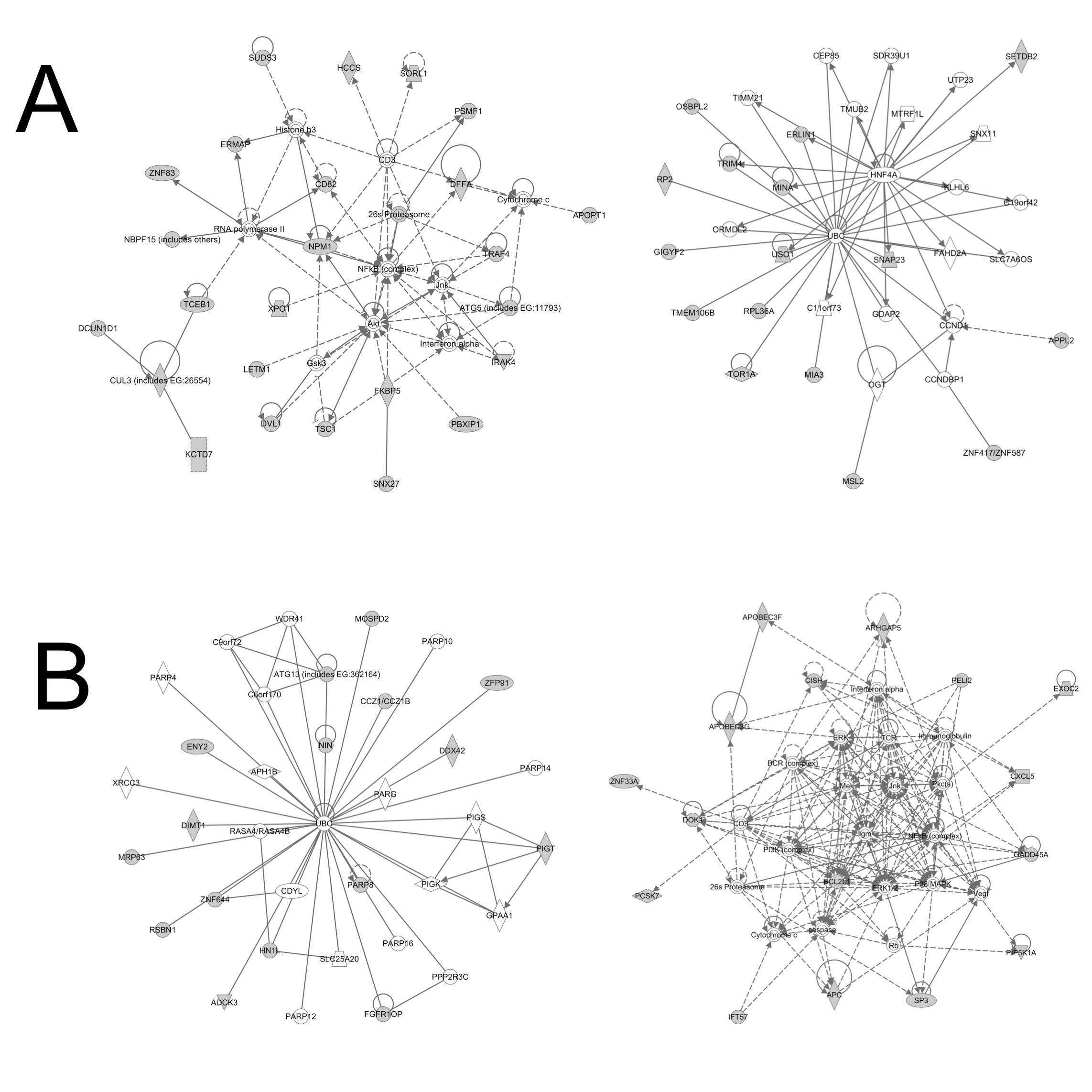


| **Short UTR Pathway Analysis** | | |
| --- | --- | --- |
| **Category** | **P value** | **# molecules** |
| Cancer | 3.2 X10^-3^ | 9 |
| Cell Cycle | 6.4 X 10^-4^ | 9 |
| Cellular Assembly | 2.2 X 10^-3^ | 13 |
