# Supplementary material for: The SLE Transcriptome Exhibits Evidence of Chronic Endotoxin Exposure and Has Widespread Dysregulation of Non-Coding and Coding RNAs
Source: PLoS One. 2014 May 5;9(5):e93846. doi: 10.1371/journal.pone.0093846 (PMC4010412; doi:10.1371/journal.pone.0093846)
Supplement: Figure S6 — Sense- antisense expression. About 5,000 coding genes had detectable antisense transcription in monocytes. A) The average transcription of antisense transcripts was less than the half of the corresponding sense transcripts; but a small number of antisense transcripts had over ten-fold higher transcription of their sense counterparts. B) The transcription of sense and antisense pairs tended to be positively correlated. The correlation between each pair was first calculated across samples in the control and SLE groups separately, then the two correlation coefficients were combined using Fisher's transformation. (DOCX) [file pone.0093846.s006.docx]

**Figure S6. Sense- antisense expression**
