# Supplementary material for: The SLE Transcriptome Exhibits Evidence of Chronic Endotoxin Exposure and Has Widespread Dysregulation of Non-Coding and Coding RNAs
Source: PLoS One. 2014 May 5;9(5):e93846. doi: 10.1371/journal.pone.0093846 (PMC4010412; doi:10.1371/journal.pone.0093846)
Supplement: Figure S7 — LncRNA association with adjacent transcription. The co-regulation of lncRNAs and their nearby coding genes was dependent on their distance and relative location. The horizontal black line is indicates the average correlation of random pairs of lncRNAs and coding genes. (Oppo: opposite strand.) (DOCX) [file pone.0093846.s007.docx]

**Figure S7. LncRNA association with adjacent transcription**
