# Supplementary material for: The SLE Transcriptome Exhibits Evidence of Chronic Endotoxin Exposure and Has Widespread Dysregulation of Non-Coding and Coding RNAs
Source: PLoS One. 2014 May 5;9(5):e93846. doi: 10.1371/journal.pone.0093846 (PMC4010412; doi:10.1371/journal.pone.0093846)
Supplement: Figure S9 — MicroRNA- target correlation. Ten pri-miRNAs and their predicted targets had a negative correlation in monocytes. The correlation was calculated using normalized read counts of pri-miRNAs and target coding genes. The correlation coefficients of each miRNA-target pair were obtained from the control and SLE groups separately and then combined using Fisher's transformation. Each bar represents the average and standard error of correlation coefficients of a target list. Target lists were downloaded from miRBase. (DOCX) [file pone.0093846.s009.docx]

**Figure S9. MicroRNA- target correlation**
