# Supplementary material for: The SLE Transcriptome Exhibits Evidence of Chronic Endotoxin Exposure and Has Widespread Dysregulation of Non-Coding and Coding RNAs
Source: PLoS One. 2014 May 5;9(5):e93846. doi: 10.1371/journal.pone.0093846 (PMC4010412; doi:10.1371/journal.pone.0093846)
Supplement: Figure S10 — miRNA validation. The increased expression of miR-212-3p in SLE was validated by qRT-PCR with cel-miR-238 as a control. Six new controls and 13 new SLE samples were used. (DOCX) [file pone.0093846.s010.docx]

**Figure S10. miRNA validation**
