# Supplementary material for: The SLE Transcriptome Exhibits Evidence of Chronic Endotoxin Exposure and Has Widespread Dysregulation of Non-Coding and Coding RNAs
Source: PLoS One. 2014 May 5;9(5):e93846. doi: 10.1371/journal.pone.0093846 (PMC4010412; doi:10.1371/journal.pone.0093846)
Supplement: Figure S11 — High expression miRNA target transcript expression. Three miRNAs whose pri-miRNAs had higher expression in SLE were examined for an effect on potential target transcripts. For these three miRNAs, target transcript expression was significantly downregulated on average. While coding genes were generally downregulated in SLE, the target genes tended to be downregulated even more. Target lists were downloaded from miRBase database. (DOCX) [file pone.0093846.s011.docx]

**Figure S11. High expression miRNA target transcript expression**
