# Supplementary material for: The SLE Transcriptome Exhibits Evidence of Chronic Endotoxin Exposure and Has Widespread Dysregulation of Non-Coding and Coding RNAs
Source: PLoS One. 2014 May 5;9(5):e93846. doi: 10.1371/journal.pone.0093846 (PMC4010412; doi:10.1371/journal.pone.0093846)
Supplement: Figure S13 — Isoforms with increased expression in SLE are more complex. The isoforms where the relative abundance was increased in SLE had a higher number of exons than those where the relative abundance was decreased (p = 0.007). On average, the SLE-favored isoforms had about 0.5 exons more than the control-favored isoforms. (DOCX) [file pone.0093846.s013.docx]

**Figure S13. Isoforms with increased expression in SLE are more complex**
