# Supplementary material for: The SLE Transcriptome Exhibits Evidence of Chronic Endotoxin Exposure and Has Widespread Dysregulation of Non-Coding and Coding RNAs
Source: PLoS One. 2014 May 5;9(5):e93846. doi: 10.1371/journal.pone.0093846 (PMC4010412; doi:10.1371/journal.pone.0093846)
Supplement: Figure S17 — Novel transcripts located on Chromosome 18. The detailed annotation of a cluster of novel transcripts located on chromosome 18 using public genomic data. A) Four novel transcripts identified by the Tophat-Cufflinks pipeline in monocytes about 20 kb upstream of coding gene SERPINB2 are shown. All four transcripts were transcribed at the opposite direction as SERPINB2, and three of them were significantly upregulated in SLE by 303% to 671% (p = 1.3E-6 to 1.5E-8). All four transcripts were validated by qPCR. B) These transcripts are not included in commonly used gene annotation databases. Two predicted genes partially overlap with these transcripts, but both are transcribed in the opposite direction. C) Sequences within this region are evolutionarily conserved. D) A very low level of transcription was detected within this region according to an ENCODE RNA-seq data set that measured transcriptomes in nine cell lines, not including monocytes. E) This region has a DNase I hypersensitive site in CD14+ monocytes according to another ENCODE data set. F) Furthermore, an ENCODE data set measuring various histone marks in CD14+ monocytes showed that this region has a nucleosome pattern indicative of active transcription. (DOCX) [file pone.0093846.s017.docx]

**Figure S17. Novel transcripts located on Chromosome 18**
