# Supplementary material for: The SLE Transcriptome Exhibits Evidence of Chronic Endotoxin Exposure and Has Widespread Dysregulation of Non-Coding and Coding RNAs
Source: PLoS One. 2014 May 5;9(5):e93846. doi: 10.1371/journal.pone.0093846 (PMC4010412; doi:10.1371/journal.pone.0093846)
Supplement: Figure S18 — Novel transcripts located on Chromosome 8. A cluster of 26 novel transcripts located on chromosome 8 within a ∼34 kb region upstream of coding gene ADAM28. A) All of the loci are transcribed in the opposite direction of ADAM28. B) None of them are included in a common gene annotation databases. Fifteen of these transcripts were significantly upregulated in SLE (p<0.01) by 80% to 3435% and none of them were downregulated in SLE. C) Most of the transcripts are located within evolutionarily conserved regions. D) ENCODE data detected very low levels of transcripts within this region in 9 cell lines. E) According to ENCODE data sets generated from monocytes, there is a DNase I hypersensitivity site within this region. F) The same sites showed a histone modification pattern of active nucleosomes. (DOCX) [file pone.0093846.s018.docx]

**Figure S18. Novel transcripts located on Chromosome 8**
