# Supplementary material for: The SLE Transcriptome Exhibits Evidence of Chronic Endotoxin Exposure and Has Widespread Dysregulation of Non-Coding and Coding RNAs
Source: PLoS One. 2014 May 5;9(5):e93846. doi: 10.1371/journal.pone.0093846 (PMC4010412; doi:10.1371/journal.pone.0093846)
Supplement: Figure S20 — Concordance of LPS, interferon and SLE gene expression. Five LPS data sets were created from four GEO monocyte data series (additional information on methods available in Methods S1). The α-interferon data set is from our previously published work. The coding genes shown to be upregulated in SLE were analyzed for upregulation after LPS stimulation and α-interferon (aIFN) treatment. A) All three LPS data sets demonstrated that LPS treatment also increased expression of the genes shown to be upregulated in SLE. N = the number of unique genes included in the analysis. B) The degree of overlap between SLE-induced genes and aIFN-induced genes was comparable to the degree of overlap between SLE-induced genes and LPS-induced genes. (DOCX) [file pone.0093846.s020.docx]

**Figure S20. Concordance of LPS, interferon and SLE gene expression**

A B
