# Supplementary material for: The SLE Transcriptome Exhibits Evidence of Chronic Endotoxin Exposure and Has Widespread Dysregulation of Non-Coding and Coding RNAs
Source: PLoS One. 2014 May 5;9(5):e93846. doi: 10.1371/journal.pone.0093846 (PMC4010412; doi:10.1371/journal.pone.0093846)
Supplement: Figure S21 — The effect of different MAP kinase inhibitors on LPS-induced novel loci expression. MonoMac6 cells (above) and primary monocytes (below) were treated with p38 (SB203580), ERK (U0126) and JNK (SP600125) inhibitors for 30 minutes and then stimulated with LPS. The y-axis represents the ratio of the LPS+ inhibitor-treated cells over the LPS alone treated cells, with the horizontal line indicating equivalence. The p38 inhibitor led to most diminished expression of the novel loci. At the right of the graph, loci 477 and 896 were included as controls, which were novel loci that were not altered in SLE. N = 3. (DOCX) [file pone.0093846.s021.docx]

**Figure S21. The effect of different MAP kinase inhibitors on LPS-induced novel loci expression**
