# Supplementary material for: The SLE Transcriptome Exhibits Evidence of Chronic Endotoxin Exposure and Has Widespread Dysregulation of Non-Coding and Coding RNAs
Source: PLoS One. 2014 May 5;9(5):e93846. doi: 10.1371/journal.pone.0093846 (PMC4010412; doi:10.1371/journal.pone.0093846)
Supplement: Figure S22 — The average of the novel loci expression. The expression levels of the novel loci, demonstrated in Figure S21, were averaged to better highlight the effect of the inhibitors. p38 (SB203580), ERK (U0126) and JNK (SP600125) inhibitors were used to define the role of this pathway on the novel loci expression. A) MonoMac6 cell results from Figure S21 were averaged to demonstrate the overall effect of the MAP kinase inhibitors. B) Similarly, in primary monocytes, the results from Figure S21 were averaged. TNF was used as a positive control for the LPS stimulation in C) MonoMac6 cells and D) primary monocytes. (DOCX) [file pone.0093846.s022.docx]

**Supplemental Figure S22. The average of the novel loci expression**

A

B

C

D
