# Supplementary material for: The SLE Transcriptome Exhibits Evidence of Chronic Endotoxin Exposure and Has Widespread Dysregulation of Non-Coding and Coding RNAs
Source: PLoS One. 2014 May 5;9(5):e93846. doi: 10.1371/journal.pone.0093846 (PMC4010412; doi:10.1371/journal.pone.0093846)
Supplement: Table S1 — Clinical characteristics of SLE patients. (DOCX) [file pone.0093846.s023.docx]

**Table S1: Clinical characteristics of SLE patients**

| **Patient** | **SLEDAI** | **Physician Estimate** | **Organ involvement (in their history)** | **Current Medications** | **Autoantibodies** |
| --- | --- | --- | --- | --- | --- |
| 1 | 7 | 0.5 | Skin, arthritis, vasculitis, serositis | Prednisone <10mg/d  Hydroxychloroquine | ANA, dsDNA, RNP, SM, ACL |
| 2 | 0 | 0.5 | Skin, arthritis | Hydroxychloroquine  NSAID | ANA, dsDNA |
| 3 | 0 | 0.5 | Arthritis | NSAID, Hydroxychloroquine | ANA, dsDNA |
| 4 | 0 | 0.5 | Skin, arthritis, serositis | Prednisone <10mg/d  Hydroxychloroquine  NSAID  Clopodogril, Azathioprine | ANA, dsDNA |
| 5 | 2 | 1.5 | Skin, arthritis, serositis, vasculitis | Prednisone <10mg/d  Hydroxychloroquine  NSAID, Azathioprine | ANA, dsDNA, ACL |
| 6 | 0 | 0.5 | Skin | Hydroxychloroquine | ANA, Ro, La, ACL |
| 7 | 0 | 0.5 | Arthritis, renal, CNS | Hydroxychloroquine  Azathioprine | ANA, ACL |
| 8 | 2 | 0 | Arthritis | NSAID | ANA, dsDNA, ACL |
| 9 | 4 | 0.5 | Skin, serositis, CNS | Prednisone <10mg/d  Hydroxychloroquine  NSAID | ANA |
