# Supplementary material for: The SLE Transcriptome Exhibits Evidence of Chronic Endotoxin Exposure and Has Widespread Dysregulation of Non-Coding and Coding RNAs
Source: PLoS One. 2014 May 5;9(5):e93846. doi: 10.1371/journal.pone.0093846 (PMC4010412; doi:10.1371/journal.pone.0093846)
Supplement: Table S2 — RNA Samples for Libraries. (DOCX) [file pone.0093846.s024.docx]

**Table S2: RNA Samples for Libraries**

| Samples | Monocytes / ml of blood | Quantity for RNA library | OD260/280 | RIN for fragmented RNA* | Total sequencing read counts | Number of aligned reads (%) | Number of non-rRNA (%) |
| --- | --- | --- | --- | --- | --- | --- | --- |
| SLE 1 | 0.075 | 1μg | 1.87 | 2.0 | 107,105,374 | 60,565,858 (56.5%) | 10,079,865 (16.6%) |
| SLE 2 | 0.100 | 1μg | 1.91 | 2.4 | 97,521,784 | 50,626,837 (51.9%) | 10,847,196 (21.4%) |
| SLE 3 | 0.057 | 1μg | 1.85 | 2.6 | 101,133,090 | 53,933,976 (53.3%) | 7,746,516 (14.3%) |
| SLE 4 | 0.068 | 1μg | 1.8 | 2.0 | 102,855,839 | 60,323,972 (58.6%) | 13,469,260 (22.3%) |
| SLE 5 | 0.102 | 1μg | 1.96 | 2.3 | 96,258,824 | 50,180,834 (52.1%) | 9,755,026 (19.4%) |
| SLE 6 | 0.088 | 1μg | 1.91 | 2.1 | 38,037,445 | 21,816,559 (57.3%) | 3,043,670 (13.9%) |
| SLE 7 | 0.056 | 1μg | 1.96 | 2.0 | 97,926,269 | 49,771,803 (50.8%) | 11,465,266 (23.0%) |
| SLE 8 | 0.166 | 1μg | 1.82 | 2.6 | 96,449,699 | 48,440,699 (50.2%) | 14,465,354 (29.8%) |
| SLE 9 | 0.130 | 1μg | 1.93 | 2.2 | 93,576,452 | 50,290,934 (53.7%) | 8,578,371 (17.0%) |
| Control 1 | 0.187 | 1μg |  | 2.2 | 104,561,219 | 55,445,139 (53.0%) | 9,688,304 (17.4%) |
| Control 2 | 0.162 | 1μg | 1.8 | 2.3 | 87,946,713 | 39,022,779 (44.3%) | 6,176,691 (15.8%) |
| Control 3 | 0.171 | 1μg | 1.8 | 2.2 | 104,770,003 | 55,544,242 (53.0%) | 11,551,464 (20.8%) |
| Control 4 | 0.157 | 1μg | 1.96 | 2.6 | 96,907,065 | 52,374,117 (54.0%) | 10,524,719 (20.1%) |
| Control 5 | 0.135 | 1μg | 1.9 | 2.4 | 101,267,410 | 53,196,797 (52.5%) | 9,089,491 (17.0%) |
| Control 6 | 0.169 | 1μg | 1.95 | 2.5 | 86,981,121 | 43,959,363 (50.5%) | 9,008,757 (20.4%) |
| Control 7 | 0.123 | 1μg | 1.98 | 2.6 | 93,129,152 | 45,478,780 (48.8%) | 7,472,003 (16.4%) |
| Control 8 | 0.153 | 1μg | 1.9 | 2.6 | 93,058,644 | 45,533,371 (48.9%) | 9,593,370 (21.0%) |
| P-value* | 0.0025 |  |  | 0.1286 | 0.6730 | 0.6058 | 0.3704 |

* The RIN values from samples prior to fragmentation were >7 but are available for only five samples.
