# Supplementary material for: The SLE Transcriptome Exhibits Evidence of Chronic Endotoxin Exposure and Has Widespread Dysregulation of Non-Coding and Coding RNAs
Source: PLoS One. 2014 May 5;9(5):e93846. doi: 10.1371/journal.pone.0093846 (PMC4010412; doi:10.1371/journal.pone.0093846)
Supplement: Table S4 — Number of differentially expressed genes/transcripts in each class. (DOCX) [file pone.0093846.s026.docx]

**Table S4:**

**Number of differentially expressed genes/transcripts in each class**

| **Class** | **Total Tested** | **Higher in SLE** | **Lower in SLE** |
| --- | --- | --- | --- |
| Known coding gene | 14501 | 324 | 329 |
| Novel loci | 8732 | 659 | 56 |
| lncRNA | 3482 | 29 | 60 |
| Small RNA | 379 | 2 | 1 |
| Repetitive element | 847 | 11 | 61 |
| Antisense transcript | 5819 | 49 | 171 |
