# Supplementary material for: The SLE Transcriptome Exhibits Evidence of Chronic Endotoxin Exposure and Has Widespread Dysregulation of Non-Coding and Coding RNAs
Source: PLoS One. 2014 May 5;9(5):e93846. doi: 10.1371/journal.pone.0093846 (PMC4010412; doi:10.1371/journal.pone.0093846)
Supplement: Table S5 — Functional categorization of differentially expressed gene by DAVID. (DOCX) [file pone.0093846.s027.docx]

**Table S5:**

**Functional categorization of differentially expressed gene by DAVID**

|  | **Gene Set** | **Count** | **Enrichment** | **P Value** | **Representative Genes** |
| --- | --- | --- | --- | --- | --- |
| **Higher in SLE** | Cytokine activity (GO:005125) | 19 | 5.67 | 6.3E-09 | *CXCL1, CXCL3, CXCL5, CCL4, CCL20, CCL22* |
|  | Immune response (GO:0006955) | 35 | 2.84 | 6.5E-08 | *C3, TNFSF14, TNFRSF4, NOTCH1* |
|  | Regulation of cell proliferation (GO:0042127) | 35 | 2.49 | 1.4E-06 | *E2F7, CSF1, CSF2, VEGFA, PLAU* |
|  | Genetic association with systemic lupus erythematosus | 12 | 3.79 | 2.2E-04 | *CXCR1, CXCR2, C3, CFB, IL1RN, IL8* |
|  | Interferon-induced 56K protein (PIRSF005680) | 3 | 31.25 | 3.5E-03 | *IFIT1, IFIT2, IFIT3* |
| **Lower in SLE** | Granules (GO:0031091) | 12 | 12.17 | 2.8E-09 | *SELP, F13A1, GP1BA, PF4, ITGB3, ITGA2B* |
|  | Cell adhesion (GO:0007155) | 28 | 2.42 | 3.4E-05 | *CDHR1, CDHR4, PCDH8, MUC4, MUC5B, MUC16* |
|  | Regulation of cell motion (GO:0051270) | 10 | 3.13 | 4.7E-03 | *BBS1, MAP3K1, VCL* |
