# Supplementary material for: The SLE Transcriptome Exhibits Evidence of Chronic Endotoxin Exposure and Has Widespread Dysregulation of Non-Coding and Coding RNAs
Source: PLoS One. 2014 May 5;9(5):e93846. doi: 10.1371/journal.pone.0093846 (PMC4010412; doi:10.1371/journal.pone.0093846)
Supplement: Table S6 — Cross-referencing of genes identified in genetic association studies and significantly upregulated in this study. (DOCX) [file pone.0093846.s028.docx]

**Table S6:**

**Cross-referencing of genes identified in genetic association studies and significantly upregulated in this study**

| **Gene** | **SLE/Control Expression** | **P Value** | **Reference** |
| --- | --- | --- | --- |
| *CFB* | 562% | 2.7E-07 | ([1](#_ENREF_1)) |
| *CYP1A1* | 267% | 9.4E-03 | ([2](#_ENREF_2)) |
| *HSPA2* | 1068% | 2.0E-05 | ([3](#_ENREF_3)) |
| *IL1RN* | 180% | 8.7E-04 | ([4](#_ENREF_4)) |
| *IL8* | 123% | 1.9E-03 | ([5](#_ENREF_5)) |
| *CXCR1/IL8RA* | 558% | 2.5E-08 | ([6](#_ENREF_6)) |
| *CXCR2/IL8RB* | 289% | 1.6E-03 | ([6](#_ENREF_6)) |
| *SPP1* | 310% | 6.4E-03 | ([7](#_ENREF_7)) |
| *SERPINE1* | 218% | 4.6E-03 | ([8](#_ENREF_8)) |
| *C3* | 206% | 2.0E-03 | ([9](#_ENREF_9)) |
| *SOD2* | 139% | 3.8E-03 | ([10](#_ENREF_10)) |
