# Supplementary material for: The SLE Transcriptome Exhibits Evidence of Chronic Endotoxin Exposure and Has Widespread Dysregulation of Non-Coding and Coding RNAs
Source: PLoS One. 2014 May 5;9(5):e93846. doi: 10.1371/journal.pone.0093846 (PMC4010412; doi:10.1371/journal.pone.0093846)
Supplement: Methods S1 — Supplemental Methods List. (DOCX) [file pone.0093846.s030.docx]

**Supplemental Methods**

## Alignment and filtering of sequencing reads

All 50bp, single-ended sequencing reads were aligned to human genome hg19 via LifeScope using the default parameters while allowing read trimming until 25bp long. Aligned reads were stored in BAM files and imported into R environment via the Rsamtools package.

Aligned reads were filtered to remove those 1) having mapping quality score (<mapq> field of SAM format) less than 10; 2) mapped to genomic loci within 100kb of assembly gaps; or 3) mapped to repetitive sequences. The remaining reads were used through the rest of this study.

## Identification of IRF1 binding sites based on sequencing depth

*Identify local maxima of sequencing depth*. All reads were extended to 200bp at the 3’-end to cover isolated regions of IRF1 binding, and then converted to sequencing depth. We assumed that the exact locations of IRF1 binding are specific to the sequence motif or have little horizontal shifting between samples, so summed up the sequencing depth of all 16 IRF1 ChIP-seq libraries for the identification of common binding sites. The accumulated depth was smoothed to remove background noise via the *filterFFT*() function of nucleR package. The 11,562,280 local maxima of the smoothed depth having accumulative depth higher than 10 were identified as potential IRF1 binding sites.

*Identify read-enriched regions from individual libraries.* The average sequencing depth within the -50bp to +50bp region around each local maximum was calculated separately for 16 IRF1ChIP-seq libraries and 4 control libraries (1 IgG ChIP and 3 input DNA controls). The resultant values were normalized by the Loess method to adjust for the difference of overall read throughput. Read-enriched regions specific to IRF1 ChIP libraries were identified from individual libraries as local maxima satisfying the following conditions. 1) Normalized depth is greater than 8.0, 2) normalized depth is significantly higher than the mean depth of four controls (p<0.01, one-sided Poisson test), 3) normalized depth is at least two fold higher than the mean depth of four controls, and 4) normalized depth is at least 50% higher than the depth of any of the 4 controls. As a result, 2,614 to 22,760 (mean = 6,065) peaks were identified from each IRF1 ChIP library.

*Identify IRF1 binding sites.* To improve specificity, only 2,134 read-enriched regions identified from two or more samples were selected as IRF1 binding sites. If two sites were located within 1kb of each other, the site having lower read enrichment related to the controls was removed. Finally, a total of 1,853 IRF1 binding sites was identified as regions having significantly higher sequencing depth in 2+ IRF1 ChIP-seq libraries than in controls.

*Estimate false discovery rate (FDR).* The FDR of the 1,853 IRF1 binding sites identified above was estimated by a permutation procedure. Each permutation randomly selected the same numbers of local maxima as the above numbers of read-enriched regions identified from each library. 100 such permutations were performed and their average numbers of selected regions found in one or more samples were summarized, based on which we estimated the FDR of the binding sites is 19.7%.

## Identification of binding sites based on both depth and IRF1 motif

Selecting binding sites solely dependent on sequencing depth might miss sites with weak IRF1 binding. Because of the strong association between binding sites and an 18bp motif we previously identified from primary monocytes using the same protocol, we identified another set of binding sites based on both sequencing depth and presence of the motif. We first selected a subset of local maxima that have a match of 80+% similarity to the 18bp motif within their -50bp to +50bp regions. We then screened this subset based on sequencing depth using the same criteria as above, but reduced the cutoff of normalized depth to 6.0 and the p value of relative enrichment to 0.05 (one-sided Poisson test). Consequently, we obtained 1,265 additional binding sites not included in the first set, making the total number of binding sites to 3,118. Both sets of binding sites were analyzed together.

## Distribution of IRF1 binding sites within human genome

Annotated regions including promoters, UTRs, exons, and introns were downloaded from RefSeq database. DNase I hypersensitivity (DHS) regions of CD14+ monocytes were downloaded from the ENCODE project. Three sets of DHS regions (one from the Crawford group and two from Stamatoyannopoulos group) were combined to get a total of 471,106 regions (with redundancy). The total length of each type of region was the number unique bases located in the regions, but more than 100kb away from any assembly gap of hg19 genome.

## Identification of sites with differential IRF1 binding in SLE

Sequencing read counts at IRF1 binding sites could be affected by both binding strength (biological) and systematic bias (technical). Systematic bias could be introduced by difference of total sequencing reads and ChIP efficiency between libraries. Normalizing for both sources involves a few parameters whose precise values are usually unknown. We assumed that most binding sites had no change in SLE and performed a conservative normalization using the Loess method after log-transformation of read counts around all 3,118 IRF1 binding sites. The normalized data was used to calculate fold change of average read counts in SLE and statistical p value with SAM test. We further used a Monte Carlo process to estimate false discovery rate (FDR). The process randomly selected the same number of local maxima with the same distribution of average read counts as the 3118 IRF1 binding sites. The same normalization and group comparison steps were performed on each of 100 such random sets. The FDR of each binding sites with given average read count and fold change was calculated as the chance of getting such site from the random sets. For example, if there were N binding sites and a total of M random sites having more than X read counts and Y fold change in SLE, the FDR was calculated as (M/100)/N.

## Integrative analysis with published data sets

## *Analysis of ENCODE from CD14+ monocytes*

The ENCODE project generated 13 sets of histone ChIP-seq data from CD14+ monocytes, including input control, CTCF, and 11 histone modifications, each with duplicated libraries (http://hgdownload.cse.ucsc.edu/goldenPath/hg19/encodeDCC/wgEncodeBroadHistone/). The BAM file of aligned reads from each library was downloaded from the UCSC ENCODE website and submitted to our BAMCHOP tool for quality control [1]. All BAM files passed quality control, except one replicate of input control that was not used in the following analysis. The average fragment length of each library was estimated by strand-strand correlation. Reads were filtered to remove those with map quality score less than 10. The remaining reads were extended at the 3’-end to 150bp long, corresponding to the 146bp nucleosome size, and shifted based on average fragment length. For example, if the estimated fragment length is 300bp, reads mapped to the forward strand will be shifted to the right by (300-150)/2=75bp whereas reads mapped to the reverse strand will shifted to the left by 75bp. Reads were then converted to sequencing depth. The sequencing depth of two duplicated libraries was average and normalized to total read count.

## *Analysis of H4ac ChIP-on-chip data*

The H4ac data generated from Affymetrix Promoter tiling array (<http://www.ncbi.nlm.nih.gov/pmc/articles/PMC2832080/>) were processed by MAT (Model-based Analysis of Tiling-array) program. Processed measurements of replicated samples (4 IgG controls, 3 no cytokine controls, and 3 for each of the cytokines) at each microarray probe were averaged and normalized to the IgG control by subtraction. IRF1 binding sites mapped to 5 or more probes within -250bp to 250bp region around them were analyzed for their H4ac pattern.

## *Analysis of transcriptome of cytokine polarization*

http://www.ncbi.nlm.nih.gov/pubmed/20625490

Expression measurements of gene level were obtained from processing of microarray data as previously described. Only genes commonly presented in different data sets were used for overlapping analysis of differentially expressed genes.

*RNA-seq data of SLE patients*

<http://www.ncbi.nlm.nih.gov/geo/query/acc.cgi?acc=GSE53419>

The processing and statistical analysis of RNA-seq data was performed previously (paper in press), and their results were used in this study for integrative analysis. Of the novel transcripts identified previously, the ones with high-novelty (no overlapping to any part of known transcripts) were overlapped to IRF1 binding sites to identify potential targets. Overlapping analysis between data sets was performed on commonly measured genes.

*DHS regions in different cell types*

[*http://ftp.ebi.ac.uk/pub/databases/ensembl/encode/integration_data_jan2011/byDataType/openchrom/jan2011/fdrPeaks/*](http://ftp.ebi.ac.uk/pub/databases/ensembl/encode/integration_data_jan2011/byDataType/openchrom/jan2011/fdrPeaks/)

DHS regions identified from 125 cell lines were downloaded as .BED files and overlapped to IRF1 binding sites. Odds ratio of IRF1 binding enrichment in DHS regions of each cell type was calculated by comparing to the overall IRF1 binding frequency within DHS regions of all 125 cell lines.

*Transcriptomic response to IRF1 overexpression*

<http://www.ncbi.nlm.nih.gov/geo/query/acc.cgi?acc=GSE26817>

Microarray data was downloaded from GEO and reprocessed to annotate data with unique gene symbols. Differential expression and false discovery rate were evaluated with SAM (Significance Analysis of Microarrays) method. Overlapping analysis of differentially expressed genes was performed on commonly measured genes.

### The primers for real time PCR

Commercially available primers were purchased from Applied Biosystems for the following genes: IFNA1 (Hs00855471_g1), IFNB1 (Hs01077958_s1), SERPINE1

(s01126606_m1), IFIH1 (Hs01070332_m1), TAP1 (Hs00388675_m1), DHX58 (Hs01597843_m1), IRF1 (Hs00971960_m1), TRIM69 (Hs00933766_m1), RARRES3 (Hs01058986_m1), IL-23A (Hs00372324_m1), IL-1B (Hs99999029_m1), ISG15 (Hs01921425_s1), TNFRSF14 (Hs00998604_m1),TNF (Hs99999043_m1), IL6 (Hs03929033_u1),GAPDH (Hs02758991_g1),TDP2 (Hs01099017_m1), KRAS (Hs00364284_g1),INHBA(Hs01081598_m1).

Primer-probe combinations for all ChIP targets were synthesized by IDT (Coralville, IA). Their sequence are listed here: TRIM69 FWD: 5’-GGAAGGAAGGAAGGGAGTAAAG-3’, TRIM69 REV: 5’-CCCACCAAACCCAGTCATAA-3’, TRIM69: 5’- /56-FAM/CCA GGAGACATTTGGCAATGACTGGA/36-TAMSp/-3’; SERPINE1 FWD: 5’-AAAGGGTAGGCAGGA GGT-3’, SERPINE1 REV: 5’-CTGCTCTTCCTCTTTGCTTCT-3’, SERPINE1 Probe: 5’-/56-FAM/TCCCTTTCTCTCTCTGAGCCTCCC /36-TAMSp/-3’; KRAS FWD: 5’-CCATCCACAT GGTCTCAGTTAT-3’, KRAS REV: 5’-GGAAAGAGAAAGTGTCTGAGGT-3’, RAS Probe: 5’-/56-FAM/TGCGTGGAACAAAT GGTTGGATGC/36-TAMSp/-3’; TDP2 FWD: 5’-TCTCTGCGGGAGGTGATAA-3’, TDP2 REV: 5’-CCCTACCTTTCC CATACCTTTC -3’, TDP2Probe: 5’-/56-FAM/AAATT GCGAGCCTTGGTCATGGC/36-TAMSp/-3’; IFIH1 FWD: 5’-GCCTGACTTTGGTT TCTGTTTC-3’, IFIH1 REV: 5’-CTTAACCAAAGGCCTCCTCTC-3’, IFIH1 Probe: 5’-//56-FAM/TGTAAACG TAATCTGCCTGGCGGG/36-TAMSp/-3’; INHBA FWD: 5’-GAGGAAGCAGGCAGGTTAAA-3’, INHBA REV: 5’-ACCAAACCAAGGAAGGA GAAG -3’, INHBA Probe: 5’-/56-FAM/TCAGCCCAGTCCTGACCAGAATCT /36-TAMSp/-3’; TNFRSF14 FWD: 5’-AGCAATGGCGCTGAGTT-3’, TNFRSF14 REV: 5’-GCCTCAAGACGTCGGTTT-3’, TNFRSF14 Probe: 5’-/56-FAM/TGCTGGAGTTCATCC TGCTAGCTG /36-TAMSp/-3’;TAP1 FWD: 5’-GCGAGAAGCTCAGCCATTTA-3’, TAP1 REV: 5’-TGA TTTCCACGCTTGCTACC-3’,TAP1 PROBE: 5’-/56-FAM/CGCCTGCTCACTAGATAACGCCT /36-TAMSp/-3’.
