# Supplementary material for: The SLE Transcriptome Exhibits Evidence of Chronic Endotoxin Exposure and Has Widespread Dysregulation of Non-Coding and Coding RNAs
Source: PLoS One. 2014 May 5;9(5):e93846. doi: 10.1371/journal.pone.0093846 (PMC4010412; doi:10.1371/journal.pone.0093846)
Supplement: References S1 — Supplemental References List: Tables. (DOCX) [file pone.0093846.s031.docx]

**References**

1. Gateva V, Sandling JK, Hom G, Taylor KE, Chung SA, Sun X, et al. A large-scale replication study identifies TNIP1, PRDM1, JAZF1, UHRF1BP1 and IL10 as risk loci for systemic lupus erythematosus. Nature Genetics. 2009;41(11):1228-33.

2. Zhang J, Deng J, Zhang C, Lu Y, Liu L, Wu Q, et al. Association of GSTT1, GSTM1 and CYP1A1 polymorphisms with susceptibility to systemic lupus erythematosus in the Chinese population. Clin Chim Acta. 2010;411(11-12):878-81.

3. Granados J, Zuniga J, Acuna-Alonzo V, Rosetti F, Vargas-Alarcon G. [Influence of alleles and haplotypes of the main histocompatibility complex on the susceptibility to systemic lupus erythematosus in the Mexican population]. Gac Med Mex. 2006;142(3):195-9.

4. Jonsen A, Gunnarsson I, Gullstrand B, Svenungsson E, Bengtsson AA, Nived O, et al. Association between SLE nephritis and polymorphic variants of the CRP and FcgammaRIIIa genes. Rheumatology. 2007;46(9):1417-21.

5. Rovin BH, Lu L, Zhang X. A novel interleukin-8 polymorphism is associated with severe systemic lupus erythematosus nephritis. Kidney Int. 2002;62(1):261-5.

6. Kato H, Tsuchiya N, Tokunaga K. Single nucleotide polymorphisms in the coding regions of human CXC-chemokine receptors CXCR1, CXCR2 and CXCR3. Genes and immunity. 2000;1(5):330-7.

7. Forton AC, Petri MA, Goldman D, Sullivan KE. An osteopontin (SPP1) polymorphism is associated with systemic lupus erythematosus. Human Mutation. 2002;On Line:496.

8. Gong R, Liu Z, Li L. Epistatic effect of plasminogen activator inhibitor 1 and beta-fibrinogen genes on risk of glomerular microthrombosis in lupus nephritis: interaction with environmental/clinical factors. Arthritis Rheum. 2007;56(5):1608-17.

9. Miyagawa H, Yamai M, Sakaguchi D, Kiyohara C, Tsukamoto H, Kimoto Y, et al. Association of polymorphisms in complement component C3 gene with susceptibility to systemic lupus erythematosus. Rheumatology. 2008;47(2):158-64.

10. Sobkowiak A, Lianeri M, Wudarski M, Lacki JK, Jagodzinski PP. Manganese superoxide dismutase Ala-9Val mitochondrial targeting sequence polymorphism in systemic lupus erythematosus in Poland. Clinical Rheumatology. 2008;27(7):827-31.
